# Supplementary material for: Prevalence of lung cancer in Colombia and a new diagnostic algorithm using health administrative databases: A real-world evidence study
Source: PLoS One. 2023 Mar 10;18(3):e0269079. doi: 10.1371/journal.pone.0269079 (PMC10004567; doi:10.1371/journal.pone.0269079)
Supplement: S1 File — (PDF) [file pone.0269079.s001.pdf]

Contents

DATA SOURCES DESCRIPTION 2
DATA ACCESS PERMISSIONS 2
DESCRIPTION OF CODE USED IN THE ANALIZES 3
Data analysis – Contributory Regime – phase I/Subjects identification 4
Data analysis – Subsidized Regime – phase I/Subjects identification 29
Data analysis – Contributory Regime – phase 2/Prevalence estimation 34
Data analysis – Subsidized Regime – phase 2/Prevalence estimation 62

DATA SOURCES DESCRIPTION

To build dabases used in this research we used the next data sources:

1. 'Unique Affiliation Database' (*Base de Datos Única de Afiliación*, or BDUA). The BDUA is the official government platform that allows the Ministry of Health to keep track on affiliation state of each individual month by month over time. This dataset contains information regarding sex, age, insurer identification and geographical information.

2. 'Study Basis for Calculation of the Capitation Unit' (*Base del Estudio de Suficiencia de la Unidad Por Capitación*, or UPC). The UPC database contains detailed records of each health service use by each Colombian enrolled in the country's health care system (including identity of the enrollee, location of service, date of service, specific type of service, any diagnostic information, identity (and type) of health professional providing the service, and payments/reimbursements for the service). This dataset have a validation process performed each time data is uploaded in order to remove non truthful data as it is the source to estimate the annual premiums paid to insurers by the government for each affiliate.

DATA ACCESS PERMISSIONS

The Clinical Research Institute of the School of Medicine at Universidad Nacional de Colombia was given access to datasets managed and maintained by the Ministry of Health of Colombia under using an anonymous identifier that allowed us to create links between databases. This agreement was emitted on March 5th, March 21st, and May 27th, 2019 and authorizes the Clinical Research Institute to carry out academic research with these databases.

Supporting file

DESCRIPTION OF CODE USED IN THE ANALIZES

For these analyzes we divided the analyzes in two phases for each year included for estimating prevalence rates. And it was also performed in two different set of databases, the contributory databases, and the subsidized databases. The figure below illustrates the process before giving detailed code.

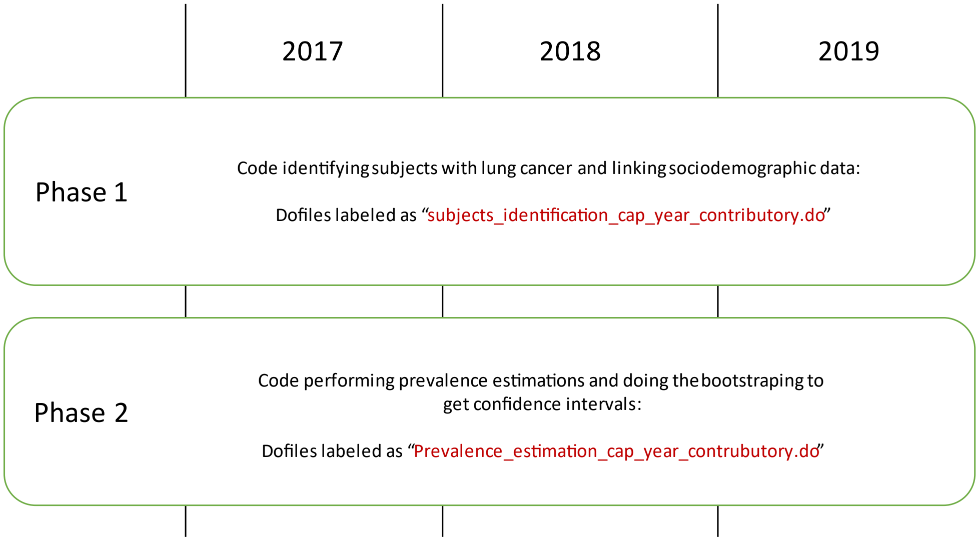

Figure 1. Data analysis process for the contributory regime

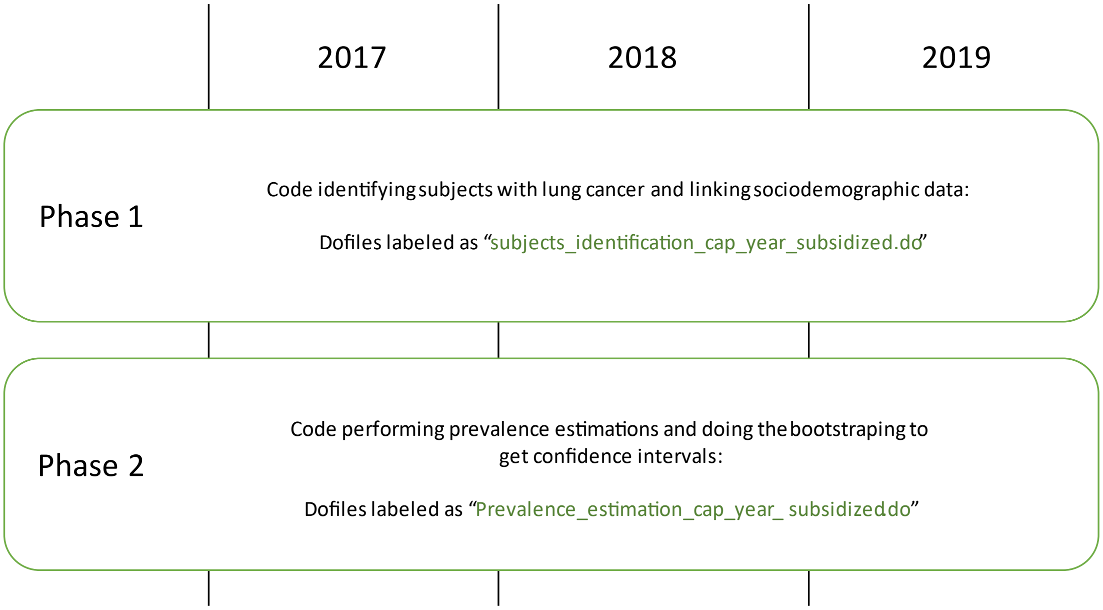

Figure 2. Data analysis process for the subsidized regime

Supporting file

Data analysis – Contributory Regime – phase I/Subjects identification

---

```
*****************************************************************************
1_subjects_identification_cap_2017_contributory
*****************************************************************************
* Opening the log file
	clear all
	*Log_files
	global logs_cap /home/javier/Documentos/Ca_pulmon/1_log_files
	*Ruta bases ORIGINALES:
	global bases_ori /home/javier/Documentos/BASES_ORI
	*Ruta bases CS:
	global bases_cap /home/javier/Documentos/Ca_pulmon/Bases_cap
	*Ruta resultados
	global resultados_cap /home/javier/Documentos/Ca_pulmon/3_Resultados/Contributivo
	*Abriendo el log
	log using "${logs_cap}/Identificacion_sujetos_cap_2017-contributivo.smcl", replace
*****************************************************************************
** Javier Amaya Nieto
*****************************************************************************


*****************************************************************************
******************************
** Estableciendo los parametros para correr la identificación de sujetos con CAP

	** Años para buscar los ID en la UPC
	local anos_ID "2015 2016 2017"
	local start_ID_year "2015"   // primer año necesita una estructura de appends diferente
	local continua_ID_years "2016 2017" // años en los que se continua haciendo el append.
	local final_ID_year "2017"   // año final en el cual se va a hacer la búsqueda de los casos
para marcar los archivos.


	** Años para buscar los servicios
	local anos_serv "2015 2016 2017"
	local start_serv_year "2015"   // primer año necesita una estructura de appends diferente
	local continua_serv_years "2016 2017" // años en los que se continua haciendo el append.
	local final_serv_year "2017"   // año final en el cual se va a hacer la búsqueda de servicios
para marcar los archivos.



*****************************************************************************
**
******** Identificando los pacientes con al menos un Dx de ca de pulmón
```

Supporting file

```
********************************************************************************
**

** Creando el loop para identificar los pacientes con el Código C34 o D381 en
 //cada BD mensual. esta identificación se hace con las BD de la UPC.
	foreach year in `anos_ID' {
	foreach month in 01 02 03 04 05 06 07 08 09 10 11 12{
	** Cargando las BD
		use "${bases_ori}/UPC/UPC`year'/upc_`year'_`month'.dta", clear

	** Creando la variable string del CIE10 para poderbuscar de a tres digitos
		gen cie10_3d=DiagnosticoCD
		recast str3 cie10_3d, force

	** Creando la variable que permite identificar a los pacientes con 1 CIE 10 DX de ca de
pulmón.
		gen ca=1 if cie10_3d=="C34" | cie10_3d=="C33" | DiagnosticoCD=="D381"

	** Dejando solo aquellos registros que tienen al menos un CIE10
		keep if ca==1

	** Colapasando la BD resultanto por ID para que quede un solo registro por mes por ID
		collapse (max) ca, by(PersonaBasicaID)

	** Guardando una BD provisional con los ID de aquellos sujetos que tuvieron al menos un
CIE10
		compress
		save "${bases_cap}/ca_id_`year'_`month'.dta", replace
	}
	}
	.


********************************************************************************
************
** Pegando todos los ID que tienen al menos un CIE10 para tene una BD consolidada

** Cargando la BD incial de enero del primer año para empezar el loop.
	use "${bases_cap}/ca_id_`start_ID_year'_01.dta", clear

** Haciendo el loop que pega todas las BD mensuales con los ID para tener un solo archivo para el
primer año
	foreach month in 02 03 04 05 06 07 08 09 10 11 12{
	append using "${bases_cap}/ca_id_`start_ID_year'_`month'.dta"
	}
	.

** Creando el loop que termina de pegar los ID de los pacientes en el periodo
```

```
	foreach year in `continua_ID_years' {
	foreach month in 01 02 03 04 05 06 07 08 09 10 11 12{
	append using "${bases_cap}/ca_id_`year'_`month'.dta"
	}
	}
	.

** Colapsando la BD final de pacientes con al menos un CIE10 para dejar una BD consolidada en el periodo seleccionado con sujetos identificados
	collapse (sum) ca, by(PersonaBasicaID)

di "----------// años seleccionados para recolectar ID de ptes con CAP: `anos_ID'"
** Unir bases de datos de servicios UPC para los años seleccionados
** Guardando la BD con los pacientes que tienen al menos un CIE 10 de ca de pulmon entre 2014-2017
	compress
	save "${bases_cap}/ca_id_`start_ID_year'-`final_ID_year'_inicial.dta", replace

****************************************************************************************************
** Pegando todos los ID que tienen al menos un CIE10 para tene una BD consolidada

** Cargando la BD incial de enero del primer año para empezar el loop.
	use "${bases_cap}/ca_id_`start_ID_year'_01.dta", clear

** Haciendo el loop que pega todas las BD mensuales con los ID para tener un solo archivo para el primer año
	foreach month in 02 03 04 05 06 07 08 09 10 11 12{
	append using "${bases_cap}/ca_id_`start_ID_year'_`month'.dta"
	}
	.

** Creando el loop que termina de pegar los ID de los pacientes en el periodo
	foreach year in `continua_ID_years' {
	foreach month in 01 02 03 04 05 06 07 08 09 10 11 12{
	append using "${bases_cap}/ca_id_`year'_`month'.dta"
	}
	}
	.

** Colapsando la BD final de pacientes con al menos un CIE10 para dejar una BD consolidada en el periodo seleccionado con sujetos identificados
	collapse (sum) ca, by(PersonaBasicaID)

di "----------// años seleccionados para recolectar ID de ptes con CAP: `anos_ID'"
** Unir bases de datos de servicios UPC para los años seleccionados
** Guardando la BD con los pacientes que tienen al menos un CIE 10 de ca de pulmon entre 2014-2017
```

```
	compress
	save "${bases_cap}/ca_id_`start_ID_year'-`final_ID_year'_inicial.dta", replace


*****************************************************************************
****************************************************
***** Creando una BD unificada con todos los servicios utilizados utilizados por los Id con ca de pulmon en el periodo elegido

** Creando el loop para hacer el merge entre la BD de sujetos identificados y las  BD de la UPC mensuales
	foreach year in `anos_serv' {
	foreach month in 01 02 03 04 05 06 07 08 09 10 11 12{
	** cargando las BD de la UPC mensuales por año y mes
		use "${bases_ori}/UPC/UPC`year'/upc_`year'_`month'.dta", clear

	** Haciendo el merge para hacer el match con lo que tienen al menos un dx de ca de pulmón
		merge m:1 PersonaBasicaID using "${bases_cap}/ca_id_`start_ID_year'-`final_ID_year'_inicial.dta"

	** Dejando solo las obseraciones que están en ambas bases de datos
		keep if _merge==3
		drop _merge

	** Arreglando la variable año de nacimiento y CUPS
		tostring AnnoNacimiento tipocodigoprocedimentocd, replace

	** Dejando solo las variables requeridas para análisis posteriores
		keep PersonaBasicaID Sexo CodigoAdministradora AnnoNacimiento MunicipioCD DiagnosticoCD FechaServicio ProcedimientoCD ambitosprocedimientocd tipocodigoprocedimentocd diasestancia ValorPagado CodigoHabilitacion ca


	*************************************************************************
*************************************************************
	** Tomando una muestra del 20% de cada BD de servicios año_mes para poderla manejar en otro pc.
		//sample 20

	** Guardando un archivo por año-mes de todos los servicios consumidos por los sujetos con al menos un dx de ca de pulmón.
		compress
		save "${bases_cap}/ca_ser_`year'_`month'.dta", replace
	}
	}
	.
```

```
di "----------// años seleccionados para recolectar servicios: `anos_serv'"

** Unir bases de datos de servicios UPC para los años seleccionados

	use "${bases_cap}/ca_ser_`start_serv_year'_01.dta", clear

	** Creando el loop para hacer append a las BD de la primera BD
	foreach month in 02 03 04 05 06 07 08 09 10 11 12{
	append using "${bases_cap}/ca_ser_`start_serv_year'_`month'.dta"
	}
	.

	** Creando el loop para hacer append a las BD de 2015 a 2017
	foreach year in `continua_serv_years' {
	foreach month in 01 02 03 04 05 06 07 08 09 10 11 12{
	append using "${bases_cap}/ca_ser_`year'_`month'.dta"
	}
	}
	.

	** Guardando la BD de servicios consolidada del periodo seleccionado con pacientes que
tienen al menos un DX CIE10 de ca de pulmón.
	compress
	save "${bases_cap}/ca_ser_`start_serv_year'-`final_serv_year'_inicial.dta", replace


***************************************************************************************
******************************
*** Ajustando variables para poder identificar la lista de pacientes con las definiciones de ca de
pulmón.
***************************************************************************************
******************************

	** Cargando la BD de todos los pacientes en el periodo que tienen al menos un código de
ca de pulmón
	use "${bases_cap}/ca_ser_`start_serv_year'-`final_serv_year'_inicial.dta", replace

	** Ajustando fecha de servicio
	gen fx_servicio=date(FechaServicio, "YMD")
	format fx_servicio %td
	gen mes_servicio=mofd(fx_servicio)
	format mes_servicio %tm
	gen year_servicio=year(fx_servicio)
	drop FechaServicio

	** Excluyendo pacientes que no consumieron servicios en el 2017 (aplicando CRITERIO DE
INCLUSIÓN EXPUESTOS# 1)---------------------------------------- INCLUSION#1
```

```
bys PersonaBasicaID: egen max_servicio=max(year_servicio)
keep if max_servicio==`final_serv_year'  // este código identifica aquellos
pacientes que consiumieron servicios en el año 2017
//** 3,455,036 observations deleted
//** quedan 6,205,101 observaciones

**drop if year_servicio == 2017 // con este código eliminamos de la estimación de
casos prevalentes de 3 años (2014,2015 y 2016) las observaciones de servicios correspondientes al
año 2017
//drop if year_servicio == 2014

** Identificando todos los servicios con CIE10 de Ca de Pulmón 2)--------------------------------
---------------------------------------------------- INCLUSION#2
gen cie10_3d=DiagnosticoCD
recast str3 cie10_3d, force
gen ca_dx=1 if cie10_3d=="C34" /*| cie10_3d=="C33" | DiagnosticoCD=="D381"*/
replace ca_dx=0 if ca_dx!=1

** Identificando CUPS de uso de servicios oncológicos 2)-------------------------------------------
------------------------------------------------- INCLUSION#2
gen       cups_ca=1 if ProcedimientoCD=="320002"
replace cups_ca=1 if ProcedimientoCD=="320003"
replace cups_ca=1 if ProcedimientoCD=="320201"
replace cups_ca=1 if ProcedimientoCD=="322800"
replace cups_ca=1 if ProcedimientoCD=="323100"
replace cups_ca=1 if ProcedimientoCD=="324101"
replace cups_ca=1 if ProcedimientoCD=="324102"
replace cups_ca=1 if ProcedimientoCD=="324200"
replace cups_ca=1 if ProcedimientoCD=="324201"
replace cups_ca=1 if ProcedimientoCD=="324202"
replace cups_ca=1 if ProcedimientoCD=="324203"
replace cups_ca=1 if ProcedimientoCD=="324204"
replace cups_ca=1 if ProcedimientoCD=="325100"
replace cups_ca=1 if ProcedimientoCD=="325101"
replace cups_ca=1 if ProcedimientoCD=="325102"
replace cups_ca=1 if ProcedimientoCD=="325200"
replace cups_ca=1 if ProcedimientoCD=="325201"
replace cups_ca=1 if ProcedimientoCD=="325202"
replace cups_ca=1 if ProcedimientoCD=="325300"
replace cups_ca=1 if ProcedimientoCD=="325301"
replace cups_ca=1 if ProcedimientoCD=="325302"
replace cups_ca=1 if ProcedimientoCD=="326101"
replace cups_ca=1 if ProcedimientoCD=="332203"
replace cups_ca=1 if ProcedimientoCD=="332204"
replace cups_ca=1 if ProcedimientoCD=="332206"
replace cups_ca=1 if ProcedimientoCD=="332207"
replace cups_ca=1 if ProcedimientoCD=="332401"
replace cups_ca=1 if ProcedimientoCD=="332500"
```

```
replace cups_ca=1 if ProcedimientoCD=="332501"
replace cups_ca=1 if ProcedimientoCD=="332601"
replace cups_ca=1 if ProcedimientoCD=="332701"
replace cups_ca=1 if ProcedimientoCD=="332702"
replace cups_ca=1 if ProcedimientoCD=="332703"
replace cups_ca=1 if ProcedimientoCD=="332704"
replace cups_ca=1 if ProcedimientoCD=="332801"
replace cups_ca=1 if ProcedimientoCD=="340200"
replace cups_ca=1 if ProcedimientoCD=="340201"
replace cups_ca=1 if ProcedimientoCD=="342100"
replace cups_ca=1 if ProcedimientoCD=="320001"
replace cups_ca=1 if ProcedimientoCD=="320202"
replace cups_ca=1 if ProcedimientoCD=="334400"
replace cups_ca=1 if ProcedimientoCD=="549004"
replace cups_ca=1 if ProcedimientoCD=="549011"
replace cups_ca=1 if ProcedimientoCD=="992501"
replace cups_ca=1 if ProcedimientoCD=="992502"
replace cups_ca=1 if ProcedimientoCD=="992503"
replace cups_ca=1 if ProcedimientoCD=="992504"
replace cups_ca=1 if ProcedimientoCD=="992505"
replace cups_ca=1 if ProcedimientoCD=="992510"
replace cups_ca=1 if ProcedimientoCD=="992506"
replace cups_ca=1 if ProcedimientoCD=="992507"
replace cups_ca=1 if ProcedimientoCD=="890287"
replace cups_ca=1 if ProcedimientoCD=="890387"
replace cups_ca=1 if ProcedimientoCD=="890487"
replace cups_ca=1 if ProcedimientoCD=="922506"
replace cups_ca=1 if ProcedimientoCD=="922800"
replace cups_ca=1 if ProcedimientoCD=="922801"
replace cups_ca=1 if ProcedimientoCD=="S22222"
replace cups_ca=0 if cups_ca!=1



*******************************************************************

************* Identificando características de base

bys PersonaBasicaID: egen residencia=mode(MunicipioCD) if  year_servicio==`final_serv_year' |
year_servicio==(`final_serv_year'-1) , maxmode // ambitosprocedimientocd=="A" &
bys PersonaBasicaID: egen eps=mode(CodigoAdministradora) if year_servicio==`final_serv_year' |
year_servicio==(`final_serv_year'-1), maxmode

gen f_nac=date(AnnoNacimiento, "YMD")
replace f_nac=date(AnnoNacimiento,"Y") if f_nac==.
format f_nac %td
drop AnnoNacimiento
```

Supporting file

```
bys PersonaBasicaID: egen sexo=mode(Sexo), maxmode

collapse (max) f_nac residencia (first) eps sexo (max) ca_dx cups_ca, by(PersonaBasicaID
mes_servicio)

collapse (max) f_nac residencia (first) eps sexo  (sum) ca_dx cups_ca, by(PersonaBasicaID)

** Sexo
gen mujer=1 if sexo=="F"
replace mujer=0 if sexo=="M"

drop if mujer==.

** Depto/Region

gen depto=int(residencia/1000)

label define depto 5 "Antioquia" 8 "Atlántico" 11 "Bogotá" 13 "Bolívar" 15 "Boyacá" 17 "Caldas" 18
"Caquetá" /*
*/ 19 "Cauca" 20 "Cesar" 23 "Córdoba" 25 "Cundinamarca" 27 "Choco" 41 "Huila" 44 "La Guajira"
47 "Magdalena" /*
*/ 50 "Meta" 52 "Nariño" 54 "N de Santander" 63 "Quindío" 66 "Risaralda" 68 "Santander" 70
"Sucre" 73 "Tolima" /*
*/ 76 "Valle del Cauca" 81 "Arauca" 85 "Casanare" 86 "Putumayo" 88 "San Andrés" 91 "Amazonas"
94 "Guainía" 95 "Guaviare" 97 "Vaupés" 99 "Vichada"

label values depto depto

gen region=1 if depto==8 | depto==13 | depto==20 | depto==23 | depto==44 | depto==47 |
depto==70 | depto==88
replace region=2 if depto==11
replace region=3 if depto==5 | depto==17 | depto==18 | depto==41 | depto==63 | depto==66 |
depto==73
replace region=4 if depto==15 | depto==25 | depto==50 | depto==54 | depto==68
replace region=5 if depto==91 | depto==81 | depto==85 | depto==86 | depto==88 | depto==94 |
depto==95 | depto==97 | depto==99
replace region=6 if depto==76 | depto==19 | depto==27 | depto==52

label define region 1 "Atlántica" 2 "Bogotá DC" 3 "Central" 4 "Oriental" 5 "Orinoquía - Amazonía" 6
"Pacífica"
label values region region


*** Definiendo grupos de edad ***

gen age=(20185-f_nac)/365.25   // fecha 20185 1 de enero de 2018
keep if age>=20 & age<=110
```

```
gen age_group = 1 if age<45
replace age_group = 2 if age>=45 & age<65
replace age_group = 3 if age>=65

label define age_group 1 "20 a 44" 2 "45 a 64" 3 "65 o más"
label values age_group age_group

************************************************************************


** Generar las definiciones

gen def_sen=1 if ca_dx>=4
gen def_esp=1 if ca_dx>=4 & cups_ca!=0


** Generando la base de datos

collapse (sum) def_sen def_esp, by(mujer region age_group)


save "${resultados_cap}/Original_estimation_Contributivo_n_3yrs_2017.dta", replace



*******************************************************************************
**************************
**Closing the log file
	log close

*****************************************************************************
2_subjects_identification_cap_2018_contributory
*****************************************************************************

* Opening the log file
	clear all
	*Log_files
	global logs_cap /home/javier/Documentos/Ca_pulmon/1_log_files
	*Ruta bases ORIGINALES:
	global bases_ori /home/javier/Documentos/BASES_ORI
	*Ruta bases CS:
	global bases_cap /home/javier/Documentos/Ca_pulmon/Bases_cap
	*Ruta resultados
	global resultados_cap /home/javier/Documentos/Ca_pulmon/3_Resultados
	*Abriendo el log
	log using "${logs_cap}/Identificacion_sujetos_cap_2018-contributivo.smcl", replace
*******************************************************************************
** Javier Amaya Nieto
```

Supporting file

```
*******************************************************************************
*****************************
** Estableciendo los parametros para correr la identificación de sujetos con CAP

	** Años para buscar los ID en la UPC
	local anos_ID "2016 2017 2018"
	local start_ID_year "2016"   // primer año necesita una estructura de appends diferente
	local continua_ID_years "2017 2018" // años en los que se continua haciendo el append.
	local final_ID_year "2018"   // año final en el cual se va a hacer la búsqueda de los casos
para marcar los archivos.


	** Años para buscar los servicios
	local anos_serv "2016 2017 2018"
	local start_serv_year "2016"   // primer año necesita una estructura de appends diferente
	local continua_serv_years "2017 2018" // años en los que se continua haciendo el append.
	local final_serv_year "2018"   // año final en el cual se va a hacer la búsqueda de servicios
para marcar los archivos.


*******************************************************************************
**
******** Identificando los pacientes con al menos un Dx de ca de pulmón
*******************************************************************************
**

** Creando el loop para identificar los pacientes con el Código C34 o D381 en
 //cada BD mensual. esta identificación se hace con las BD de la UPC.
	foreach year in `anos_ID' {
	foreach month in 01 02 03 04 05 06 07 08 09 10 11 12{
	** Cargando las BD
		use "${bases_ori}/UPC/UPC`year'/upc_`year'_`month'.dta", clear

	** Creando la variable string del CIE10 para poderbuscar de a tres digitos
		gen cie10_3d=DiagnosticoCD
		recast str3 cie10_3d, force

	** Creando la variable que permite identificar a los pacientes con 1 CIE 10 DX de ca de
pulmón.
		gen ca=1 if cie10_3d=="C34" | cie10_3d=="C33" | DiagnosticoCD=="D381"

	** Dejando solo aquellos registros que tienen al menos un CIE10
		keep if ca==1

	** Colapasando la BD resultanto por ID para que quede un solo registro por mes por ID
		collapse (max) ca, by(PersonaBasicaID)
```

```
        ** Guardando una BD provisional con los ID de aquellos sujetos que tuvieron al menos un
CIE10
                compress
                save "${bases_cap}/ca_id_`year'_`month'.dta", replace
        }
        }
        .


**********************************************************************************
************
** Pegando todos los ID que tienen al menos un CIE10 para tene una BD consolidada

** Cargando la BD incial de enero del primer año para empezar el loop.
        use "${bases_cap}/ca_id_`start_ID_year'_01.dta", clear

** Haciendo el loop que pega todas las BD mensuales con los ID para tener un solo archivo para el
primer año
        foreach month in 02 03 04 05 06 07 08 09 10 11 12{
        append using "${bases_cap}/ca_id_`start_ID_year'_`month'.dta"
        }
        .

** Creando el loop que termina de pegar los ID de los pacientes en el periodo
        foreach year in `continua_ID_years' {
        foreach month in 01 02 03 04 05 06 07 08 09 10 11 12{
        append using "${bases_cap}/ca_id_`year'_`month'.dta"
        }
        }
        .

** Colapsando la BD final de pacientes con al menos un CIE10 para dejar una BD consolidada en el
periodo seleccionado con sujetos identificados
        collapse (sum) ca, by(PersonaBasicaID)

di "----------// años seleccionados para recolectar ID de ptes con CAP: `anos_ID'"
** Unir bases de datos de servicios UPC para los años seleccionados
** Guardando la BD con los pacientes que tienen al menos un CIE 10 de ca de pulmon entre 2014-
2017
        compress
        save "${bases_cap}/ca_id_`start_ID_year'-`final_ID_year'_inicial.dta", replace

**********************************************************************************
************
** Pegando todos los ID que tienen al menos un CIE10 para tene una BD consolidada

** Cargando la BD incial de enero del primer año para empezar el loop.
```

```
	use "${bases_cap}/ca_id_`start_ID_year'_01.dta", clear

** Haciendo el loop que pega todas las BD mensuales con los ID para tener un solo archivo para el primer año
	foreach month in 02 03 04 05 06 07 08 09 10 11 12{
	append using "${bases_cap}/ca_id_`start_ID_year'_`month'.dta"
	}
	.

** Creando el loop que termina de pegar los ID de los pacientes en el periodo
	foreach year in `continua_ID_years' {
	foreach month in 01 02 03 04 05 06 07 08 09 10 11 12{
	append using "${bases_cap}/ca_id_`year'_`month'.dta"
	}
	}
	.

** Colapsando la BD final de pacientes con al menos un CIE10 para dejar una BD consolidada en el periodo seleccionado con sujetos identificados
	collapse (sum) ca, by(PersonaBasicaID)

di "----------// años seleccionados para recolectar ID de ptes con CAP: `anos_ID'"
** Unir bases de datos de servicios UPC para los años seleccionados
** Guardando la BD con los pacientes que tienen al menos un CIE 10 de ca de pulmon entre 2014-2017
	compress
	save "${bases_cap}/ca_id_`start_ID_year'-`final_ID_year'_inicial.dta", replace


****************************************************************************************************************************************
***** Creando una BD unificada con todos los servicios utilizados utilizados por los Id con ca de pulmon en el periodo elegido

** Creando el loop para hacer el merge entre la BD de sujetos identificados y las  BD de la UPC mensuales
	foreach year in `anos_serv' {
	foreach month in 01 02 03 04 05 06 07 08 09 10 11 12{
	** cargando las BD de la UPC mensuales por año y mes
		use "${bases_ori}/UPC/UPC`year'/upc_`year'_`month'.dta", clear

	** Haciendo el merge para hacer el match con lo que tienen al menos un dx de ca de pulmón
		merge m:1 PersonaBasicaID using "${bases_cap}/ca_id_`start_ID_year'-`final_ID_year'_inicial.dta"

	** Dejando solo las obseraciones que están en ambas bases de datos
		keep if _merge==3
```

```
		drop _merge

	** Arreglando la variable año de nacimiento y CUPS
		tostring AnnoNacimiento , replace

	** Dejando solo las variables requeridas para análisis posteriores
		keep PersonaBasicaID Sexo CodigoAdministradora AnnoNacimiento MunicipioCD DiagnosticoCD FechaServicio ProcedimientoCD ambitosprocedimientocd diasestancia ValorPagado CodigoHabilitacion ca


	*****************************************************************************************************************************
	** Tomando una muestra del 20% de cada BD de servicios año_mes para poderla manejar en otro pc.
		//sample 20

	** Guardando un archivo por año-mes de todos los servicios consumidos por los sujetos con al menos un dx de ca de pulmón.
		compress
		save "${bases_cap}/ca_ser_`year'_`month'.dta", replace
	}
	}
	.


di "----------// años seleccionados para recolectar servicios: `anos_serv'"

** Unir bases de datos de servicios UPC para los años seleccionados

	use "${bases_cap}/ca_ser_`start_serv_year'_01.dta", clear

	** Creando el loop para hacer append a las BD de la primera BD
	foreach month in 02 03 04 05 06 07 08 09 10 11 12{
	append using "${bases_cap}/ca_ser_`start_serv_year'_`month'.dta"
	}
	.

	** Creando el loop para hacer append a las BD de 2015 a 2017
	foreach year in `continua_serv_years' {
	foreach month in 01 02 03 04 05 06 07 08 09 10 11 12{
	append using "${bases_cap}/ca_ser_`year'_`month'.dta"
	}
	}
	.

	** Guardando la BD de servicios consolidada del periodo seleccionado con pacientes que tienen al menos un DX CIE10 de ca de pulmón.
```

```
	compress
	save "${bases_cap}/ca_ser_`start_serv_year'-`final_serv_year'_inicial.dta", replace

****************************************************************************
**************************
*** Ajustando variables para poder identificar la lista de pacientes con las definiciones de ca de pulmón.
****************************************************************************
**************************

	** Cargando la BD de todos los pacientes en el periodo que tienen al menos un código de ca de pulmón
	use "${bases_cap}/ca_ser_`start_serv_year'-`final_serv_year'_inicial.dta", replace

	** Ajustando fecha de servicio
	gen fx_servicio=date(FechaServicio, "YMD")
	format fx_servicio %td
	gen mes_servicio=mofd(fx_servicio)
	format mes_servicio %tm
	gen year_servicio=year(fx_servicio)
	drop FechaServicio

	** Excluyendo pacientes que no consumieron servicios en el 2017 (aplicando CRITERIO DE INCLUSIÓN EXPUESTOS# 1)------------------------------------------ INCLUSION#1
		bys PersonaBasicaID: egen max_servicio=max(year_servicio)
		keep if max_servicio==`final_serv_year'  // este código identifica aquellos pacientes que consiumieron servicios en el año 2017
		//** 3,455,036 observations deleted
		//** quedan 6,205,101 observaciones

		**drop if year_servicio == 2017 // con este código eliminamos de la estimación de casos prevalentes de 3 años (2014,2015 y 2016) las observaciones de servicios correspondientes al año 2017
		//drop if year_servicio == 2014

	** Identificando todos los servicios con CIE10 de Ca de Pulmón 2)---------------------------------
---------------------------------------------------- INCLUSION#2
		gen cie10_3d=DiagnosticoCD
		recast str3 cie10_3d, force
		gen ca_dx=1 if cie10_3d=="C34" /*| cie10_3d=="C33" | DiagnosticoCD=="D381"*/
		replace ca_dx=0 if ca_dx!=1

	** Identificando CUPS de uso de servicios oncológicos 2)---------------------------------------------
------------------------------------------------- INCLUSION#2
		gen	cups_ca=1 if ProcedimientoCD=="320002"
		replace cups_ca=1 if ProcedimientoCD=="320003"
		replace cups_ca=1 if ProcedimientoCD=="320201"
		replace cups_ca=1 if ProcedimientoCD=="322800"
```

Supporting file

```
replace cups_ca=1 if ProcedimientoCD=="323100"
replace cups_ca=1 if ProcedimientoCD=="324101"
replace cups_ca=1 if ProcedimientoCD=="324102"
replace cups_ca=1 if ProcedimientoCD=="324200"
replace cups_ca=1 if ProcedimientoCD=="324201"
replace cups_ca=1 if ProcedimientoCD=="324202"
replace cups_ca=1 if ProcedimientoCD=="324203"
replace cups_ca=1 if ProcedimientoCD=="324204"
replace cups_ca=1 if ProcedimientoCD=="325100"
replace cups_ca=1 if ProcedimientoCD=="325101"
replace cups_ca=1 if ProcedimientoCD=="325102"
replace cups_ca=1 if ProcedimientoCD=="325200"
replace cups_ca=1 if ProcedimientoCD=="325201"
replace cups_ca=1 if ProcedimientoCD=="325202"
replace cups_ca=1 if ProcedimientoCD=="325300"
replace cups_ca=1 if ProcedimientoCD=="325301"
replace cups_ca=1 if ProcedimientoCD=="325302"
replace cups_ca=1 if ProcedimientoCD=="326101"
replace cups_ca=1 if ProcedimientoCD=="332203"
replace cups_ca=1 if ProcedimientoCD=="332204"
replace cups_ca=1 if ProcedimientoCD=="332206"
replace cups_ca=1 if ProcedimientoCD=="332207"
replace cups_ca=1 if ProcedimientoCD=="332401"
replace cups_ca=1 if ProcedimientoCD=="332500"
replace cups_ca=1 if ProcedimientoCD=="332501"
replace cups_ca=1 if ProcedimientoCD=="332601"
replace cups_ca=1 if ProcedimientoCD=="332701"
replace cups_ca=1 if ProcedimientoCD=="332702"
replace cups_ca=1 if ProcedimientoCD=="332703"
replace cups_ca=1 if ProcedimientoCD=="332704"
replace cups_ca=1 if ProcedimientoCD=="332801"
replace cups_ca=1 if ProcedimientoCD=="340200"
replace cups_ca=1 if ProcedimientoCD=="340201"
replace cups_ca=1 if ProcedimientoCD=="342100"
replace cups_ca=1 if ProcedimientoCD=="320001"
replace cups_ca=1 if ProcedimientoCD=="320202"
replace cups_ca=1 if ProcedimientoCD=="334400"
replace cups_ca=1 if ProcedimientoCD=="549004"
replace cups_ca=1 if ProcedimientoCD=="549011"
replace cups_ca=1 if ProcedimientoCD=="992501"
replace cups_ca=1 if ProcedimientoCD=="992502"
replace cups_ca=1 if ProcedimientoCD=="992503"
replace cups_ca=1 if ProcedimientoCD=="992504"
replace cups_ca=1 if ProcedimientoCD=="992505"
replace cups_ca=1 if ProcedimientoCD=="992510"
replace cups_ca=1 if ProcedimientoCD=="992506"
replace cups_ca=1 if ProcedimientoCD=="992507"
replace cups_ca=1 if ProcedimientoCD=="890287"
```

```
replace cups_ca=1 if ProcedimientoCD=="890387"
replace cups_ca=1 if ProcedimientoCD=="890487"
replace cups_ca=1 if ProcedimientoCD=="922506"
replace cups_ca=1 if ProcedimientoCD=="922800"
replace cups_ca=1 if ProcedimientoCD=="922801"
replace cups_ca=1 if ProcedimientoCD=="S22222"
replace cups_ca=0 if cups_ca!=1




*******************************************************************

************* Identificando características de base

bys PersonaBasicaID: egen residencia=mode(MunicipioCD) if  year_servicio==`final_serv_year' |
year_servicio==(`final_serv_year'-1), maxmode // ambitosprocedimientocd=="A" &
bys PersonaBasicaID: egen eps=mode(CodigoAdministradora) if year_servicio==`final_serv_year' |
year_servicio==(`final_serv_year'-1), maxmode

gen f_nac=date(AnnoNacimiento, "YMD")
replace f_nac=date(AnnoNacimiento,"Y") if f_nac==.
format f_nac %td
drop AnnoNacimiento

bys PersonaBasicaID: egen sexo=mode(Sexo), maxmode

collapse (max) f_nac residencia (first) eps sexo (max) ca_dx cups_ca, by(PersonaBasicaID
mes_servicio)

collapse (max) f_nac residencia (first) eps sexo  (sum) ca_dx cups_ca, by(PersonaBasicaID)

** Sexo
gen mujer=1 if sexo=="F"
replace mujer=0 if sexo=="M"

drop if mujer==.

** Depto/Region

gen depto=int(residencia/1000)

label define depto 5 "Antioquia" 8 "Atlántico" 11 "Bogotá" 13 "Bolívar" 15 "Boyacá" 17 "Caldas" 18
"Caquetá" /*
*/ 19 "Cauca" 20 "Cesar" 23 "Córdoba" 25 "Cundinamarca" 27 "Choco" 41 "Huila" 44 "La Guajira"
47 "Magdalena" /*
*/ 50 "Meta" 52 "Nariño" 54 "N de Santander" 63 "Quindío" 66 "Risaralda" 68 "Santander" 70
"Sucre" 73 "Tolima" /*
```

```
*/ 76 "Valle del Cauca" 81 "Arauca" 85 "Casanare" 86 "Putumayo" 88 "San Andrés" 91 "Amazonas"
94 "Guainía" 95 "Guaviare" 97 "Vaupés" 99 "Vichada"

label values depto depto

gen region=1 if depto==8 | depto==13 | depto==20 | depto==23 | depto==44 | depto==47 |
depto==70 | depto==88
replace region=2 if depto==11
replace region=3 if depto==5 | depto==17 | depto==18 | depto==41 | depto==63 | depto==66 |
depto==73
replace region=4 if depto==15 | depto==25 | depto==50 | depto==54 | depto==68
replace region=5 if depto==91 | depto==81 | depto==85 | depto==86 | depto==88 | depto==94 |
depto==95 | depto==97 | depto==99
replace region=6 if depto==76 | depto==19 | depto==27 | depto==52

label define region 1 "Atlántica" 2 "Bogotá DC" 3 "Central" 4 "Oriental" 5 "Orinoquía - Amazonía" 6
"Pacífica"
label values region region


*** Definiendo grupos de edad ***

gen age=(`final_serv_year'5-f_nac)/365.25   // fecha 20185 1 de enero de 2018
keep if age>=20 & age<=110
gen age_group = 1 if age<45
replace age_group = 2 if age>=45 & age<65
replace age_group = 3 if age>=65

label define age_group 1 "20 a 44" 2 "45 a 64" 3 "65 o más"
label values age_group age_group

**********************************************************************


** Generar las definiciones

gen def_sen=1 if ca_dx>=4
gen def_esp=1 if ca_dx>=4 & cups_ca!=0


** Generando la base de datos

collapse (sum) def_sen def_esp, by(mujer region age_group)


save "${resultados_cap}/Original_estimation_Contributivo_n_3yrs_`final_serv_year'.dta", replace
```

Supporting file

****
****
**Closing the log file
	log close

****
3_subjects_identification_cap_2019_contributory
****

* Opening the log file
	clear all
	*Log_files
	global logs_cap /home/javier/Documentos/Ca_pulmon/1_log_files
	*Ruta bases ORIGINALES:
	global bases_ori /home/javier/Documentos/BASES_ORI
	*Ruta bases CS:
	global bases_cap /home/javier/Documentos/Ca_pulmon/Bases_cap
	*Ruta resultados
	global resultados_cap /home/javier/Documentos/Ca_pulmon/3_Resultados
	*Abriendo el log
	log using "${logs_cap}/Identificacion_sujetos_cap_2019-contributivo.smcl", replace
****
** Javier Amaya Nieto

****
****
** Estableciendo los parametros para correr la identificación de sujetos con CAP

	** Años para buscar los ID en la UPC
	local anos_ID "2017 2018 2019"
	local start_ID_year "2017" // primer año necesita una estructura de appends diferente
	local continua_ID_years "2018 2019" // años en los que se continua haciendo el append.
	local final_ID_year "2019" // año final en el cual se va a hacer la búsqueda de los casos para marcar los archivos.

	** Años para buscar los servicios
	local anos_serv "2017 2018 2019"
	local start_serv_year "2017" // primer año necesita una estructura de appends diferente
	local continua_serv_years "2018 2019" // años en los que se continua haciendo el append.
	local final_serv_year "2019" // año final en el cual se va a hacer la búsqueda de servicios para marcar los archivos.

Supporting file

```
********************************************************************************
**
******** Identificando los pacientes con al menos un Dx de ca de pulmón
********************************************************************************
**

** Creando el loop para identificar los pacientes con el Código C34 o D381 en
 //cada BD mensual. esta identificación se hace con las BD de la UPC.
	foreach year in `anos_ID' {
	foreach month in 01 02 03 04 05 06 07 08 09 10 11 12{
	** Cargando las BD
		use "${bases_ori}/UPC/UPC`year'/upc_`year'_`month'.dta", clear

	** Creando la variable string del CIE10 para poderbuscar de a tres digitos
		gen cie10_3d=DiagnosticoCD
		recast str3 cie10_3d, force

	** Creando la variable que permite identificar a los pacientes con 1 CIE 10 DX de ca de
pulmón.
		gen ca=1 if cie10_3d=="C34" | cie10_3d=="C33" | DiagnosticoCD=="D381"

	** Dejando solo aquellos registros que tienen al menos un CIE10
		keep if ca==1

	** Colapasando la BD resultanto por ID para que quede un solo registro por mes por ID
		collapse (max) ca, by(PersonaBasicaID)

	** Guardando una BD provisional con los ID de aquellos sujetos que tuvieron al menos un
CIE10
		compress
		save "${bases_cap}/ca_id_`year'_`month'.dta", replace
	}
	}
	.


********************************************************************************
************
** Pegando todos los ID que tienen al menos un CIE10 para tene una BD consolidada

** Cargando la BD incial de enero del primer año para empezar el loop.
	use "${bases_cap}/ca_id_`start_ID_year'_01.dta", clear

** Haciendo el loop que pega todas las BD mensuales con los ID para tener un solo archivo para el
primer año
	foreach month in 02 03 04 05 06 07 08 09 10 11 12{
	append using "${bases_cap}/ca_id_`start_ID_year'_`month'.dta"
	}
```

```
.

** Creando el loop que termina de pegar los ID de los pacientes en el periodo
	foreach year in `continua_ID_years' {
	foreach month in 01 02 03 04 05 06 07 08 09 10 11 12{
	append using "${bases_cap}/ca_id_`year'_`month'.dta"
	}
	}
	.

** Colapsando la BD final de pacientes con al menos un CIE10 para dejar una BD consolidada en el periodo seleccionado con sujetos identificados
	collapse (sum) ca, by(PersonaBasicaID)

di "----------// años seleccionados para recolectar ID de ptes con CAP: `anos_ID'"
** Unir bases de datos de servicios UPC para los años seleccionados
** Guardando la BD con los pacientes que tienen al menos un CIE 10 de ca de pulmon entre 2014-2017
	compress
	save "${bases_cap}/ca_id_`start_ID_year'-`final_ID_year'_inicial.dta", replace

************************************************************************************
** Pegando todos los ID que tienen al menos un CIE10 para tene una BD consolidada

** Cargando la BD incial de enero del primer año para empezar el loop.
	use "${bases_cap}/ca_id_`start_ID_year'_01.dta", clear

** Haciendo el loop que pega todas las BD mensuales con los ID para tener un solo archivo para el primer año
	foreach month in 02 03 04 05 06 07 08 09 10 11 12{
	append using "${bases_cap}/ca_id_`start_ID_year'_`month'.dta"
	}
	.

** Creando el loop que termina de pegar los ID de los pacientes en el periodo
	foreach year in `continua_ID_years' {
	foreach month in 01 02 03 04 05 06 07 08 09 10 11 12{
	append using "${bases_cap}/ca_id_`year'_`month'.dta"
	}
	}
	.

** Colapsando la BD final de pacientes con al menos un CIE10 para dejar una BD consolidada en el periodo seleccionado con sujetos identificados
	collapse (sum) ca, by(PersonaBasicaID)

di "----------// años seleccionados para recolectar ID de ptes con CAP: `anos_ID'"
```

Supporting file

```
** Unir bases de datos de servicios UPC para los años seleccionados
** Guardando la BD con los pacientes que tienen al menos un CIE 10 de ca de pulmon entre 2014-2017
	compress
	save "${bases_cap}/ca_id_`start_ID_year'-`final_ID_year'_inicial.dta", replace


*********************************************************************************************************************************
***** Creando una BD unificada con todos los servicios utilizados utilizados por los Id con ca de pulmon en el periodo elegido

** Creando el loop para hacer el merge entre la BD de sujetos identificados y las  BD de la UPC mensuales
	foreach year in `anos_serv' {
	foreach month in 01 02 03 04 05 06 07 08 09 10 11 12{
	** cargando las BD de la UPC mensuales por año y mes
		use "${bases_ori}/UPC/UPC`year'/upc_`year'_`month'.dta", clear

	** Haciendo el merge para hacer el match con lo que tienen al menos un dx de ca de pulmón
		merge m:1 PersonaBasicaID using "${bases_cap}/ca_id_`start_ID_year'-`final_ID_year'_inicial.dta"

	** Dejando solo las obseraciones que están en ambas bases de datos
		keep if _merge==3
		drop _merge

	** Arreglando la variable año de nacimiento y CUPS
		tostring AnnoNacimiento , replace

	** Dejando solo las variables requeridas para análisis posteriores
		keep PersonaBasicaID Sexo CodigoAdministradora AnnoNacimiento MunicipioCD DiagnosticoCD FechaServicio ProcedimientoCD ambitosprocedimientocd diasestancia ValorPagado CodigoHabilitacion ca


	*********************************************************************************************************************************
	** Tomando una muestra del 20% de cada BD de servicios año_mes para poderla manejar en otro pc.
		//sample 20

	** Guardando un archivo por año-mes de todos los servicios consumidos por los sujetos con al menos un dx de ca de pulmón.
		compress
		save "${bases_cap}/ca_ser_`year'_`month'.dta", replace
	}
```

```
            }
            .


di "----------// años seleccionados para recolectar servicios: `anos_serv'"

** Unir bases de datos de servicios UPC para los años seleccionados

            use "${bases_cap}/ca_ser_`start_serv_year'_01.dta", clear

            ** Creando el loop para hacer append a las BD de la primera BD
            foreach month in 02 03 04 05 06 07 08 09 10 11 12{
            append using "${bases_cap}/ca_ser_`start_serv_year'_`month'.dta"
            }
            .

            ** Creando el loop para hacer append a las BD de 2015 a 2017
            foreach year in `continua_serv_years' {
            foreach month in 01 02 03 04 05 06 07 08 09 10 11 12{
            append using "${bases_cap}/ca_ser_`year'_`month'.dta"
            }
            }
            .

            ** Guardando la BD de servicios consolidada del periodo seleccionado con pacientes que
tienen al menos un DX CIE10 de ca de pulmón.
            compress
            save "${bases_cap}/ca_ser_`start_serv_year'-`final_serv_year'_inicial.dta", replace


*******************************************************************************
**************************
*** Ajustando variables para poder identificar la lista de pacientes con las definiciones de ca de
pulmón.
*******************************************************************************
**************************

            ** Cargando la BD de todos los pacientes en el periodo que tienen al menos un código de
ca de pulmón
            use "${bases_cap}/ca_ser_`start_serv_year'-`final_serv_year'_inicial.dta", replace

            ** Ajustando fecha de servicio
            gen fx_servicio=date(FechaServicio, "YMD")
            format fx_servicio %td
            gen mes_servicio=mofd(fx_servicio)
            format mes_servicio %tm
            gen year_servicio=year(fx_servicio)
            drop FechaServicio
```

Supporting file

```
** Excluyendo pacientes que no consumieron servicios en el 2017 (aplicando CRITERIO DE INCLUSIÓN EXPUESTOS# 1)------------------------------------------ INCLUSION#1
		bys PersonaBasicaID: egen max_servicio=max(year_servicio)
		keep if max_servicio==`final_serv_year'  // este código identifica aquellos pacientes que consiumieron servicios en el año 2017
		//** 3,455,036 observations deleted
		//** quedan 6,205,101 observaciones

		**drop if year_servicio == 2017 // con este código eliminamos de la estimación de casos prevalentes de 3 años (2014,2015 y 2016) las observaciones de servicios correspondientes al año 2017
		//drop if year_servicio == 2014

** Identificando todos los servicios con CIE10 de Ca de Pulmón 2)---------------------------------------------------------------------------------------- INCLUSION#2
		gen cie10_3d=DiagnosticoCD
		recast str3 cie10_3d, force
		gen ca_dx=1 if cie10_3d=="C34" /*| cie10_3d=="C33" | DiagnosticoCD=="D381"*/
		replace ca_dx=0 if ca_dx!=1

** Identificando CUPS de uso de servicios oncológicos 2)-------------------------------------------------------------------------------------------- INCLUSION#2
		gen	cups_ca=1 if ProcedimientoCD=="320002"
		replace cups_ca=1 if ProcedimientoCD=="320003"
		replace cups_ca=1 if ProcedimientoCD=="320201"
		replace cups_ca=1 if ProcedimientoCD=="322800"
		replace cups_ca=1 if ProcedimientoCD=="323100"
		replace cups_ca=1 if ProcedimientoCD=="324101"
		replace cups_ca=1 if ProcedimientoCD=="324102"
		replace cups_ca=1 if ProcedimientoCD=="324200"
		replace cups_ca=1 if ProcedimientoCD=="324201"
		replace cups_ca=1 if ProcedimientoCD=="324202"
		replace cups_ca=1 if ProcedimientoCD=="324203"
		replace cups_ca=1 if ProcedimientoCD=="324204"
		replace cups_ca=1 if ProcedimientoCD=="325100"
		replace cups_ca=1 if ProcedimientoCD=="325101"
		replace cups_ca=1 if ProcedimientoCD=="325102"
		replace cups_ca=1 if ProcedimientoCD=="325200"
		replace cups_ca=1 if ProcedimientoCD=="325201"
		replace cups_ca=1 if ProcedimientoCD=="325202"
		replace cups_ca=1 if ProcedimientoCD=="325300"
		replace cups_ca=1 if ProcedimientoCD=="325301"
		replace cups_ca=1 if ProcedimientoCD=="325302"
		replace cups_ca=1 if ProcedimientoCD=="326101"
		replace cups_ca=1 if ProcedimientoCD=="332203"
		replace cups_ca=1 if ProcedimientoCD=="332204"
		replace cups_ca=1 if ProcedimientoCD=="332206"
```

```
replace cups_ca=1 if ProcedimientoCD=="332207"
replace cups_ca=1 if ProcedimientoCD=="332401"
replace cups_ca=1 if ProcedimientoCD=="332500"
replace cups_ca=1 if ProcedimientoCD=="332501"
replace cups_ca=1 if ProcedimientoCD=="332601"
replace cups_ca=1 if ProcedimientoCD=="332701"
replace cups_ca=1 if ProcedimientoCD=="332702"
replace cups_ca=1 if ProcedimientoCD=="332703"
replace cups_ca=1 if ProcedimientoCD=="332704"
replace cups_ca=1 if ProcedimientoCD=="332801"
replace cups_ca=1 if ProcedimientoCD=="340200"
replace cups_ca=1 if ProcedimientoCD=="340201"
replace cups_ca=1 if ProcedimientoCD=="342100"
replace cups_ca=1 if ProcedimientoCD=="320001"
replace cups_ca=1 if ProcedimientoCD=="320202"
replace cups_ca=1 if ProcedimientoCD=="334400"
replace cups_ca=1 if ProcedimientoCD=="549004"
replace cups_ca=1 if ProcedimientoCD=="549011"
replace cups_ca=1 if ProcedimientoCD=="992501"
replace cups_ca=1 if ProcedimientoCD=="992502"
replace cups_ca=1 if ProcedimientoCD=="992503"
replace cups_ca=1 if ProcedimientoCD=="992504"
replace cups_ca=1 if ProcedimientoCD=="992505"
replace cups_ca=1 if ProcedimientoCD=="992510"
replace cups_ca=1 if ProcedimientoCD=="992506"
replace cups_ca=1 if ProcedimientoCD=="992507"
replace cups_ca=1 if ProcedimientoCD=="890287"
replace cups_ca=1 if ProcedimientoCD=="890387"
replace cups_ca=1 if ProcedimientoCD=="890487"
replace cups_ca=1 if ProcedimientoCD=="922506"
replace cups_ca=1 if ProcedimientoCD=="922800"
replace cups_ca=1 if ProcedimientoCD=="922801"
replace cups_ca=1 if ProcedimientoCD=="S22222"
replace cups_ca=0 if cups_ca!=1



*****************************************************************

************* Identificando características de base

bys PersonaBasicaID: egen residencia=mode(MunicipioCD) if  year_servicio==`final_serv_year' |
year_servicio==(`final_serv_year'-1), maxmode // ambitosprocedimientocd=="A" &
bys PersonaBasicaID: egen eps=mode(CodigoAdministradora) if year_servicio==`final_serv_year' |
year_servicio==(`final_serv_year'-1), maxmode

gen f_nac=date(AnnoNacimiento, "YMD")
```

Supporting file

```
replace f_nac=date(AnnoNacimiento,"Y") if f_nac==.
format f_nac %td
drop AnnoNacimiento

bys PersonaBasicaID: egen sexo=mode(Sexo), maxmode

collapse (max) f_nac residencia (first) eps sexo (max) ca_dx cups_ca, by(PersonaBasicaID
mes_servicio)

collapse (max) f_nac residencia (first) eps sexo  (sum) ca_dx cups_ca, by(PersonaBasicaID)

** Sexo
gen mujer=1 if sexo=="F"
replace mujer=0 if sexo=="M"

drop if mujer==.

** Depto/Region

gen depto=int(residencia/1000)

label define depto 5 "Antioquia" 8 "Atlántico" 11 "Bogotá" 13 "Bolívar" 15 "Boyacá" 17 "Caldas" 18
"Caquetá" /*
*/ 19 "Cauca" 20 "Cesar" 23 "Córdoba" 25 "Cundinamarca" 27 "Choco" 41 "Huila" 44 "La Guajira"
47 "Magdalena" /*
*/ 50 "Meta" 52 "Nariño" 54 "N de Santander" 63 "Quindío" 66 "Risaralda" 68 "Santander" 70
"Sucre" 73 "Tolima" /*
*/ 76 "Valle del Cauca" 81 "Arauca" 85 "Casanare" 86 "Putumayo" 88 "San Andrés" 91 "Amazonas"
94 "Guainía" 95 "Guaviare" 97 "Vaupés" 99 "Vichada"

label values depto depto

gen region=1 if depto==8 | depto==13 | depto==20 | depto==23 | depto==44 | depto==47 |
depto==70 | depto==88
replace region=2 if depto==11
replace region=3 if depto==5 | depto==17 | depto==18 | depto==41 | depto==63 | depto==66 |
depto==73
replace region=4 if depto==15 | depto==25 | depto==50 | depto==54 | depto==68
replace region=5 if depto==91 | depto==81 | depto==85 | depto==86 | depto==88 | depto==94 |
depto==95 | depto==97 | depto==99
replace region=6 if depto==76 | depto==19 | depto==27 | depto==52

label define region 1 "Atlántica" 2 "Bogotá DC" 3 "Central" 4 "Oriental" 5 "Orinoquía - Amazonía" 6
"Pacífica"
label values region region


*** Definiendo grupos de edad ***
```

```
gen age=(`final_serv_year'5-f_nac)/365.25   // fecha `final_serv_year'5 1 de enero del último año evaluado
keep if age>=20 & age<=110
gen age_group = 1 if age<45
replace age_group = 2 if age>=45 & age<65
replace age_group = 3 if age>=65

label define age_group 1 "20 a 44" 2 "45 a 64" 3 "65 o más"
label values age_group age_group

*********************************************************************


** Generar las definiciones

gen def_sen=1 if ca_dx>=4
gen def_esp=1 if ca_dx>=4 & cups_ca!=0


** Generando la base de datos

collapse (sum) def_sen def_esp, by(mujer region age_group)


save "${resultados_cap}/Original_estimation_Contributivo_n_3yrs_`final_serv_year'.dta", replace


*****************************************************************************
**************************
**Closing the log file
	log close
```

Data analysis – Subsidized Regime – phase I/Subjects identification

****

1_subjects_identification_cap_2017_subsidized

```
***************************************************************************
* Opening the log file
	clear all
	*Log_files
```

Supporting file

```
global logs_cap /home/javier/Documentos/Ca_pulmon/1_log_files
*Ruta bases ORIGINALES:
global bases_ori /home/javier/Documentos/BASES_ORI
*Ruta bases CS:
global bases_cap /home/javier/Documentos/Ca_pulmon/Bases_cap
*Ruta resultados
global resultados_cap /home/javier/Documentos/Ca_pulmon/3_Resultados/Subsidiado
*Abriendo el log
log using "${logs_cap}/Identificacion_sujetos_cap_2017-Subsidiado.smcl", replace

********************************************************************************
** Lung cancer Project-Javier Amaya Nieto


********************************************************************************
******************************
** Estableciendo los parametros para correr la identificación de sujetos con CAP

** Años para buscar los ID en la UPC
local anos_ID "2014 2015 2016"
local start_ID_year "2014"   // primer año necesita una estructura de appends diferente
local continua_ID_years "2015 2016" // años en los que se continua haciendo el append.
local final_ID_year "2016"   // año final en el cual se va a hacer la búsqueda de los casos para marcar los archivos.


** Años para buscar los servicios
local anos_serv "2014 2015 2016"
local start_serv_year "2014"   // primer año necesita una estructura de appends diferente
local continua_serv_years "2015 2016" // años en los que se continua haciendo el append.
local final_serv_year "2016"   // año final en el cual se va a hacer la búsqueda de servicios para marcar los archivos.

foreach year in `anos_ID' {
use "${bases_ori}/UPC/SDS_C_Salud/Capital_UPC_`year'.dta", clear
gen cie10_3d=cie10
recast str3 cie10_3d, force
gen ca=1 if cie10_3d=="C34"
keep if ca==1
collapse (max) ca, by(id)
save "${bases_cap}/CS_ca_id_`year'_CS.dta", replace
}
** collapse ID
use "${bases_cap}/CS_ca_id_`start_ID_year'_CS.dta", clear
foreach year in `continua_ID_years' {
append using "${bases_cap}/CS_ca_id_`year'_CS.dta"
}
collapse (sum) ca, by(id)
```

```
save "${bases_cap}/CS_ca_id_`start_ID_year'-`final_ID_year'_CS.dta", replace

** Seleccion de servicios utilizados por los Id entre ene-2014 y dic-2017
foreach year in `anos_serv' {
use "${bases_ori}/UPC/SDS_C_Salud/Capital_UPC_`year'.dta", clear
merge n:1 id using "${bases_cap}/CS_ca_id_`start_ID_year'-`final_ID_year'_CS.dta"
keep if _merge==3
drop _merge
tostring fecha_nac tipo_cups, replace
save "${bases_cap}/CS_ca_ser_`year'_CS.dta", replace
}

** Unir bases de datos de servicios de salud para
use "${bases_cap}/CS_ca_ser_`start_serv_year'_CS.dta", clear

foreach year in `continua_serv_years' {
append using "${bases_cap}/CS_ca_ser_`year'_CS.dta"
}

save "${bases_cap}/CS_ca_ser_`start_serv_year'-`final_serv_year'_CS.dta", replace

*******************************************************************************
**********
log close
```

****

2_subjects_identification_cap_2018_subsidized

****

```
* Opening the log file
	clear all
	*Log_files
	global logs_cap /home/javier/Documentos/Ca_pulmon/1_log_files
	*Ruta bases ORIGINALES:
	global bases_ori /home/javier/Documentos/BASES_ORI
	*Ruta bases CS:
	global bases_cap /home/javier/Documentos/Ca_pulmon/Bases_cap
	*Ruta resultados
	global resultados_cap /home/javier/Documentos/Ca_pulmon/3_Resultados/Subsidiado
	*Abriendo el log
	log using "${logs_cap}/Identificacion_sujetos_cap_2018-Subsidiado.smcl", replace

*******************************************************************************
** Lung cancer Project-Javier Amaya Nieto


*******************************************************************************
*****************************
** Estableciendo los parametros para correr la identificación de sujetos con CAP
```

Supporting file

```
** Años para buscar los ID en la UPC
local anos_ID "2015 2016 2017"
local start_ID_year "2015"   // primer año necesita una estructura de appends diferente
local continua_ID_years "2016 2017" // años en los que se continua haciendo el append.
local final_ID_year "2017"   // año final en el cual se va a hacer la búsqueda de los casos
para marcar los archivos.


** Años para buscar los servicios
local anos_serv "2015 2016 2017"
local start_serv_year "2015"   // primer año necesita una estructura de appends diferente
local continua_serv_years "2016 2017" // años en los que se continua haciendo el append.
local final_serv_year "2017"   // año final en el cual se va a hacer la búsqueda de servicios
para marcar los archivos.

foreach year in `anos_ID' {
use "${bases_ori}/UPC/SDS_C_Salud/Capital_UPC_`year'.dta", clear
gen cie10_3d=cie10
recast str3 cie10_3d, force
gen ca=1 if cie10_3d=="C34"
keep if ca==1
collapse (max) ca, by(id)
save "${bases_cap}/CS_ca_id_`year'_CS.dta", replace
}
** collapse ID
use "${bases_cap}/CS_ca_id_`start_ID_year'_CS.dta", clear
foreach year in `continua_ID_years' {
append using "${bases_cap}/CS_ca_id_`year'_CS.dta"
}
collapse (sum) ca, by(id)
save "${bases_cap}/CS_ca_id_`start_ID_year'-`final_ID_year'_CS.dta", replace

** Seleccion de servicios utilizados por los Id entre ene-2014 y dic-2017
foreach year in `anos_serv' {
use "${bases_ori}/UPC/SDS_C_Salud/Capital_UPC_`year'.dta", clear
merge n:1 id using "${bases_cap}/CS_ca_id_`start_ID_year'-`final_ID_year'_CS.dta"
keep if _merge==3
drop _merge
tostring fecha_nac tipo_cups, replace
save "${bases_cap}/CS_ca_ser_`year'_CS.dta", replace
}

** Unir bases de datos de servicios de salud para
use "${bases_cap}/CS_ca_ser_`start_serv_year'_CS.dta", clear

foreach year in `continua_serv_years' {
append using "${bases_cap}/CS_ca_ser_`year'_CS.dta"
```

}

save "${bases_cap}/CS_ca_ser_`start_serv_year'-`final_serv_year'_CS.dta", replace

****
****
log close

****
3_subjects_identification_cap_2019_subsidized
****
* Opening the log file
    clear all
    *Log_files
    global logs_cap /home/javier/Documentos/Ca_pulmon/1_log_files
    *Ruta bases ORIGINALES:
    global bases_ori /home/javier/Documentos/BASES_ORI
    *Ruta bases CS:
    global bases_cap /home/javier/Documentos/Ca_pulmon/Bases_cap
    *Ruta resultados
    global resultados_cap /home/javier/Documentos/Ca_pulmon/3_Resultados/Subsidiado
    *Abriendo el log
    log using "${logs_cap}/Identificacion_sujetos_cap_2019-Subsidiado.smcl", replace

****
** Lung cancer Project-Javier Amaya Nieto

****
****
** Estableciendo los parametros para correr la identificación de sujetos con CAP

    ** Años para buscar los ID en la UPC
    local anos_ID "2016 2017 2018"
    local start_ID_year "2016"   // primer año necesita una estructura de appends diferente
    local continua_ID_years "2017 2018" // años en los que se continua haciendo el append.
    local final_ID_year "2018"   // año final en el cual se va a hacer la búsqueda de los casos
para marcar los archivos.

    ** Años para buscar los servicios
    local anos_serv "2016 2017 2018"
    local start_serv_year "2016"   // primer año necesita una estructura de appends diferente
    local continua_serv_years "2017 2018" // años en los que se continua haciendo el append.
    local final_serv_year "2018"   // año final en el cual se va a hacer la búsqueda de servicios
para marcar los archivos.

foreach year in `anos_ID' {

```
use "${bases_ori}/UPC/SDS_C_Salud/Capital_UPC_`year'.dta", clear
gen cie10_3d=cie10
recast str3 cie10_3d, force
gen ca=1 if cie10_3d=="C34"
keep if ca==1
collapse (max) ca, by(id)
save "${bases_cap}/CS_ca_id_`year'_CS.dta", replace
}
** collapse ID
use "${bases_cap}/CS_ca_id_`start_ID_year'_CS.dta", clear
foreach year in `continua_ID_years' {
append using "${bases_cap}/CS_ca_id_`year'_CS.dta"
}
collapse (sum) ca, by(id)
save "${bases_cap}/CS_ca_id_`start_ID_year'-`final_ID_year'_CS.dta", replace

** Seleccion de servicios utilizados por los Id entre ene-2014 y dic-2017
foreach year in `anos_serv' {
use "${bases_ori}/UPC/SDS_C_Salud/Capital_UPC_`year'.dta", clear
merge n:1 id using "${bases_cap}/CS_ca_id_`start_ID_year'-`final_ID_year'_CS.dta"
keep if _merge==3
drop _merge
tostring fecha_nac tipo_cups, replace
save "${bases_cap}/CS_ca_ser_`year'_CS.dta", replace
}

** Unir bases de datos de servicios de salud para
use "${bases_cap}/CS_ca_ser_`start_serv_year'_CS.dta", clear

foreach year in `continua_serv_years' {
append using "${bases_cap}/CS_ca_ser_`year'_CS.dta"
}

save "${bases_cap}/CS_ca_ser_`start_serv_year'-`final_serv_year'_CS.dta", replace

*****************************************************************************
**********
log close
```

Data analysis – Contributory Regime – phase 2/Prevalence estimation

****
3_Prevalence_estimation_cap_2017_contributory

Supporting file

```
*************************************************************************

* Opening the log file
	clear all
	*Log_files
	global logs_cap /home/javier/Documentos/Ca_pulmon/1_log_files
	*Ruta bases ORIGINALES:
	global bases_ori /home/javier/Documentos/BASES_ORI
	*Ruta bases CS:
	global bases_cap /home/javier/Documentos/Ca_pulmon/Bases_cap
	*Ruta resultados
	global resultados_cap /home/javier/Documentos/Ca_pulmon/3_Resultados/Contributivo
	*Abriendo el log
	log using "${logs_cap}/Prevalence_estimation_2017-contributivo_bootstrapping.smcl",
replace

***************************************************************************
****************************
**# Preparación de las bases para el bootstrapping



** Estableciendo los parametros para correr la identificación de sujetos con CAP

	** Años para buscar los ID en la UPC
	local anos_ID "2015 2016 2017"
	local start_ID_year "2015"   // primer año necesita una estructura de appends diferente
	local continua_ID_years "2016 2017" // años en los que se continua haciendo el append.
	local final_ID_year "2017"   // año final en el cual se va a hacer la búsqueda de los casos
para marcar los archivos.


	** Años para buscar los servicios
	local anos_serv "2015 2016 2017"
	local start_serv_year "2015"   // primer año necesita una estructura de appends diferente
	local continua_serv_years "2016 2017" // años en los que se continua haciendo el append.
	local final_serv_year "2017"   // año final en el cual se va a hacer la búsqueda de servicios
para marcar los archivos.



foreach samp of numlist 1(1)1000 {


	** Cargando la BD de todos los pacientes en el periodo que tienen al menos un código de
ca de pulmón
	use "${bases_cap}/ca_ser_`start_serv_year'-`final_serv_year'_inicial.dta", replace
```

```
	** Ajustando fecha de servicio
	gen fx_servicio=date(FechaServicio, "YMD")
	format fx_servicio %td
	gen mes_servicio=mofd(fx_servicio)
	format mes_servicio %tm
	gen year_servicio=year(fx_servicio)
	drop FechaServicio

	** Excluyendo pacientes que no consumieron servicios en el 2017 (aplicando CRITERIO DE INCLUSIÓN EXPUESTOS# 1)----------------------------------------- INCLUSION#1
		bys PersonaBasicaID: egen max_servicio=max(year_servicio)
		keep if max_servicio==`final_serv_year'  // este código identifica aquellos pacientes que consiumieron servicios en el año 2017
		//** 3,455,036 observations deleted
		//** quedan 6,205,101 observaciones

		**drop if year_servicio == 2017 // con este código eliminamos de la estimación de casos prevalentes de 3 años (2014,2015 y 2016) las observaciones de servicios correspondientes al año 2017
		//drop if year_servicio == 2014

	** Identificando todos los servicios con CIE10 de Ca de Pulmón 2)----------------------------------------------------------------------------------------------------- INCLUSION#2
		gen cie10_3d=DiagnosticoCD
		recast str3 cie10_3d, force
		gen ca_dx=1 if cie10_3d=="C34" /*| cie10_3d=="C33" | DiagnosticoCD=="D381"*/
		replace ca_dx=0 if ca_dx!=1

	** Identificando CUPS de uso de servicios oncológicos 2)------------------------------------------------------------------------------------------------------------- INCLUSION#2
		gen 	cups_ca=1 if ProcedimientoCD=="320002"
		replace cups_ca=1 if ProcedimientoCD=="320003"
		replace cups_ca=1 if ProcedimientoCD=="320201"
		replace cups_ca=1 if ProcedimientoCD=="322800"
		replace cups_ca=1 if ProcedimientoCD=="323100"
		replace cups_ca=1 if ProcedimientoCD=="324101"
		replace cups_ca=1 if ProcedimientoCD=="324102"
		replace cups_ca=1 if ProcedimientoCD=="324200"
		replace cups_ca=1 if ProcedimientoCD=="324201"
		replace cups_ca=1 if ProcedimientoCD=="324202"
		replace cups_ca=1 if ProcedimientoCD=="324203"
		replace cups_ca=1 if ProcedimientoCD=="324204"
		replace cups_ca=1 if ProcedimientoCD=="325100"
		replace cups_ca=1 if ProcedimientoCD=="325101"
		replace cups_ca=1 if ProcedimientoCD=="325102"
		replace cups_ca=1 if ProcedimientoCD=="325200"
		replace cups_ca=1 if ProcedimientoCD=="325201"
		replace cups_ca=1 if ProcedimientoCD=="325202"
```

```
replace cups_ca=1 if ProcedimientoCD=="325300"
replace cups_ca=1 if ProcedimientoCD=="325301"
replace cups_ca=1 if ProcedimientoCD=="325302"
replace cups_ca=1 if ProcedimientoCD=="326101"
replace cups_ca=1 if ProcedimientoCD=="332203"
replace cups_ca=1 if ProcedimientoCD=="332204"
replace cups_ca=1 if ProcedimientoCD=="332206"
replace cups_ca=1 if ProcedimientoCD=="332207"
replace cups_ca=1 if ProcedimientoCD=="332401"
replace cups_ca=1 if ProcedimientoCD=="332500"
replace cups_ca=1 if ProcedimientoCD=="332501"
replace cups_ca=1 if ProcedimientoCD=="332601"
replace cups_ca=1 if ProcedimientoCD=="332701"
replace cups_ca=1 if ProcedimientoCD=="332702"
replace cups_ca=1 if ProcedimientoCD=="332703"
replace cups_ca=1 if ProcedimientoCD=="332704"
replace cups_ca=1 if ProcedimientoCD=="332801"
replace cups_ca=1 if ProcedimientoCD=="340200"
replace cups_ca=1 if ProcedimientoCD=="340201"
replace cups_ca=1 if ProcedimientoCD=="342100"
replace cups_ca=1 if ProcedimientoCD=="320001"
replace cups_ca=1 if ProcedimientoCD=="320202"
replace cups_ca=1 if ProcedimientoCD=="334400"
replace cups_ca=1 if ProcedimientoCD=="549004"
replace cups_ca=1 if ProcedimientoCD=="549011"
replace cups_ca=1 if ProcedimientoCD=="992501"
replace cups_ca=1 if ProcedimientoCD=="992502"
replace cups_ca=1 if ProcedimientoCD=="992503"
replace cups_ca=1 if ProcedimientoCD=="992504"
replace cups_ca=1 if ProcedimientoCD=="992505"
replace cups_ca=1 if ProcedimientoCD=="992510"
replace cups_ca=1 if ProcedimientoCD=="992506"
replace cups_ca=1 if ProcedimientoCD=="992507"
replace cups_ca=1 if ProcedimientoCD=="890287"
replace cups_ca=1 if ProcedimientoCD=="890387"
replace cups_ca=1 if ProcedimientoCD=="890487"
replace cups_ca=1 if ProcedimientoCD=="922506"
replace cups_ca=1 if ProcedimientoCD=="922800"
replace cups_ca=1 if ProcedimientoCD=="922801"
replace cups_ca=1 if ProcedimientoCD=="S22222"
replace cups_ca=0 if cups_ca!=1
```

** Identificando características de base

```
bys PersonaBasicaID: egen residencia=mode(MunicipioCD) if  year_servicio==`final_serv_year' | year_servicio==(`final_serv_year'-1), maxmode // ambitosprocedimientocd=="A" &
bys PersonaBasicaID: egen eps=mode(CodigoAdministradora) if year_servicio==`final_serv_year' | year_servicio==(`final_serv_year'-1), maxmode
```

Supporting file

```
gen f_nac=date(AnnoNacimiento, "YMD")
replace f_nac=date(AnnoNacimiento,"Y") if f_nac==.
format f_nac %td
drop AnnoNacimiento

bys PersonaBasicaID: egen sexo=mode(Sexo), maxmode

collapse (max) f_nac residencia (first) eps sexo (max) ca_dx cups_ca, by(PersonaBasicaID
mes_servicio)

collapse (max) f_nac residencia (first) eps sexo  (sum) ca_dx cups_ca, by(PersonaBasicaID)



***//////////////////////////////////////////////////////////////////////////////////////////////
////
**********************************************************************************
*************************
*** Linea para hacer el bootstraping

bsample, cluster(PersonaBasicaID) idcluster(PersonaBasicaID_s)

**********************************************************************************
**************************
***//////////////////////////////////////////////////////////////////////////////////////////////
////



** Sexo
gen mujer=1 if sexo=="F"
replace mujer=0 if sexo=="M"

drop if mujer==.

** Depto/Region

gen depto=int(residencia/1000)

label define depto 5 "Antioquia" 8 "Atlántico" 11 "Bogotá" 13 "Bolívar" 15 "Boyacá" 17 "Caldas" 18
"Caquetá" /*
*/ 19 "Cauca" 20 "Cesar" 23 "Córdoba" 25 "Cundinamarca" 27 "Choco" 41 "Huila" 44 "La Guajira"
47 "Magdalena" /*
*/ 50 "Meta" 52 "Nariño" 54 "N de Santander" 63 "Quindío" 66 "Risaralda" 68 "Santander" 70
"Sucre" 73 "Tolima" /*
```

```
*/ 76 "Valle del Cauca" 81 "Arauca" 85 "Casanare" 86 "Putumayo" 88 "San Andrés" 91 "Amazonas"
94 "Guainía" 95 "Guaviare" 97 "Vaupés" 99 "Vichada"

label values depto depto

gen region=1 if depto==8 | depto==13 | depto==20 | depto==23 | depto==44 | depto==47 |
depto==70 | depto==88
replace region=2 if depto==11
replace region=3 if depto==5 | depto==17 | depto==18 | depto==41 | depto==63 | depto==66 |
depto==73
replace region=4 if depto==15 | depto==25 | depto==50 | depto==54 | depto==68
replace region=5 if depto==91 | depto==81 | depto==85 | depto==86 | depto==88 | depto==94 |
depto==95 | depto==97 | depto==99
replace region=6 if depto==76 | depto==19 | depto==27 | depto==52

label define region 1 "Atlántica" 2 "Bogotá DC" 3 "Central" 4 "Oriental" 5 "Orinoquía - Amazonía" 6
"Pacífica"
label values region region


*** Definiendo grupos de edad ***

gen age=(`final_serv_year'5-f_nac)/365.25   // fecha 20185 1 de enero de 2018
keep if age>=20 & age<=110
gen age_group = 1 if age<45
replace age_group = 2 if age>=45 & age<65
replace age_group = 3 if age>=65

label define age_group 1 "20 a 44" 2 "45 a 64" 3 "65 o más"
label values age_group age_group

***********************************************************************


** Generar las definiciones

gen def_sen=1 if ca_dx>=4
gen def_esp=1 if ca_dx>=4 & cups_ca!=0


** Generando la base de datos

collapse (sum) def_sen def_esp, by(mujer region age_group)


save
"${resultados_cap}/bootstrapping/boots_Contributivo_n_3yrs_`final_serv_year'_rep`samp'.dta",
replace
```

```
}



********************************************************************************
******
**# Prevalencia por SEXO
********************************************************************************
*******

foreach samp of numlist 1(1)1000 {

* Cargando la base
use "${resultados_cap}/bootstrapping/boots_Contributivo_n_3yrs_2017_rep`samp'.dta", clear

********************************************************************************
*******
** Estimando la prevalencia por sexo y grupo etario

merge 1:1 mujer region age_group using
"/home/javier/Documentos/Ca_pulmon/Bases_cap/Denominadores_BDUA.dta"
drop _merge

collapse (sum) def_sen def_esp denominador_2017, by(mujer)

gen cum_deno= sum(denominador_2017)
egen tot_denominador_2017 = max(cum_deno)
drop cum_deno

gen cumsen= sum(def_sen)
gen cumesp= sum(def_esp)

egen tot_sen = max(cumsen)
egen tot_esp = max(cumesp)

drop cumsen cumesp

gen p_str_sen= (def_sen/denominador_2017)*100000
gen p_str_esp= (def_esp/denominador_2017)*100000

gen p_total_sen = (tot_sen/tot_denominador_2017)*100000
gen p_total_esp = (tot_esp/tot_denominador_2017)*100000


save "${resultados_cap}/bootstrapping/Pre_estimates_sex_2017_rep`samp'.dta", replace
```

```
}


** Uniendo las estimaciones del bootstraping para poder sacar las medidas agregadas:


use "${resultados_cap}/bootstrapping/Pre_estimates_sex_2017_rep1.dta", clear

foreach samp of numlist 2(1)1000 {

append using "${resultados_cap}/bootstrapping/Pre_estimates_sex_2017_rep`samp'.dta"

}

** Haciendo las estimaciones de las prevalencias específicas y sensibles por estratos (sexo y edad)

table ( mujer) (), statistic(mean p_str_sen )  statistic(p25 p_str_sen) statistic(p75 p_str_sen)
statistic(mean p_str_esp )  statistic(p25 p_str_esp) statistic(p75 p_str_esp) nototals
collect label levels mujer 0 "Hombre" 1 "Mujer", modify
collect export "${resultados_cap}/Prevalencias_estrato_sexo_2017.docx", as(docx) replace

*******************************************************************************
******
**# Prevalencia por GRUPO ETARIO
*******************************************************************************
*******

foreach samp of numlist 1(1)1000 {

* Cargando la base
use "${resultados_cap}/bootstrapping/boots_Contributivo_n_3yrs_2017_rep`samp'.dta", clear

*******************************************************************************
*******
** Estimando la prevalencia por sexo y grupo etario

merge 1:1 mujer region age_group using
"/home/javier/Documentos/Ca_pulmon/Bases_cap/Denominadores_BDUA.dta"
drop _merge

collapse (sum) def_sen def_esp denominador_2017, by(age_group)

gen cum_deno= sum(denominador_2017)
egen tot_denominador_2017 = max(cum_deno)
drop cum_deno

gen cumsen= sum(def_sen)
gen cumesp= sum(def_esp)
```

```
egen tot_sen = max(cumsen)
egen tot_esp = max(cumesp)

drop cumsen cumesp

gen p_str_sen= (def_sen/denominador_2017)*100000
gen p_str_esp= (def_esp/denominador_2017)*100000

gen p_total_sen = (tot_sen/tot_denominador_2017)*100000
gen p_total_esp = (tot_esp/tot_denominador_2017)*100000


save "${resultados_cap}/bootstrapping/Pre_estimates_age_2017_rep`samp'.dta", replace

}


** Uniendo las estimaciones del bootstraping para poder sacar las medidas agregadas:


use "${resultados_cap}/bootstrapping/Pre_estimates_age_2017_rep1.dta", clear

foreach samp of numlist 2(1)1000 {

append using "${resultados_cap}/bootstrapping/Pre_estimates_age_2017_rep`samp'.dta"

}

** Haciendo las estimaciones de las prevalencias específicas y sensibles por estratos (sexo y edad)

table (age_group) (), statistic(mean p_str_sen )  statistic(p25 p_str_sen) statistic(p75 p_str_sen)
statistic(mean p_str_esp )  statistic(p25 p_str_esp) statistic(p75 p_str_esp) nototals
collect export "${resultados_cap}/Prevalencias_estrato_edad_2017.docx", as(docx) replace

*****************************************************************************
******
**# Prevalencia por REGION
*****************************************************************************
*******

foreach samp of numlist 1(1)1000 {

* Cargando la base
use "${resultados_cap}/bootstrapping/boots_Contributivo_n_3yrs_2017_rep`samp'.dta", clear

*****************************************************************************
*******
```

Supporting file

```
** Estimando la prevalencia por sexo y grupo etario

merge 1:1 mujer region age_group using
"/home/javier/Documentos/Ca_pulmon/Bases_cap/Denominadores_BDUA.dta"
drop _merge

collapse (sum) def_sen def_esp denominador_2017, by(region)

gen cum_deno= sum(denominador_2017)
egen tot_denominador_2017 = max(cum_deno)
drop cum_deno

gen cumsen= sum(def_sen)
gen cumesp= sum(def_esp)

egen tot_sen = max(cumsen)
egen tot_esp = max(cumesp)

drop cumsen cumesp

gen p_str_sen= (def_sen/denominador_2017)*100000
gen p_str_esp= (def_esp/denominador_2017)*100000

gen p_total_sen = (tot_sen/tot_denominador_2017)*100000
gen p_total_esp = (tot_esp/tot_denominador_2017)*100000


save "${resultados_cap}/bootstrapping/Pre_estimates_region_2017_rep`samp'.dta", replace

}


** Uniendo las estimaciones del bootstraping para poder sacar las medidas agregadas:


use "${resultados_cap}/bootstrapping/Pre_estimates_region_2017_rep1.dta", clear

foreach samp of numlist 2(1)1000 {

append using "${resultados_cap}/bootstrapping/Pre_estimates_region_2017_rep`samp'.dta"

}

** Haciendo las estimaciones de las prevalencias específicas y sensibles por estratos (REGION)

table ( region) (), statistic(mean p_str_sen )  statistic(p25 p_str_sen) statistic(p75 p_str_sen)
statistic(mean p_str_esp )  statistic(p25 p_str_esp) statistic(p75 p_str_esp) nototals
collect export "${resultados_cap}/Prevalencias_estrato_region_2017.docx", as(docx) replace
```

** Haciendo la tabla de prevalencia general
table () (), statistic(mean p_str_sen) statistic(p25 p_str_sen) statistic(p75 p_str_sen) statistic(mean p_str_esp) statistic(p25 p_str_esp) statistic(p75 p_str_esp) nototals
collect export "${resultados_cap}/Prevalencias_total_2017.docx", as(docx) replace

****
log close

****
5_Prevalence_estimation_cap_2018_contributory
****

```
* Opening the log file
	clear all
	*Log_files
	global logs_cap /home/javier/Documentos/Ca_pulmon/1_log_files
	*Ruta bases ORIGINALES:
	global bases_ori /home/javier/Documentos/BASES_ORI
	*Ruta bases CS:
	global bases_cap /home/javier/Documentos/Ca_pulmon/Bases_cap
	*Ruta resultados
	global resultados_cap /home/javier/Documentos/Ca_pulmon/3_Resultados/Contributivo
	*Abriendo el log
	log using "${logs_cap}/Prevalence_estimation_2018-contributivo_bootstrapping.smcl", replace

*******************************************************************************************************
**# Preparación de las bases para el bootstrapping

** Estableciendo los parametros para correr la identificación de sujetos con CAP

	** Años para buscar los ID en la UPC
	local anos_ID "2016 2017 2018"
	local start_ID_year "2016"   // primer año necesita una estructura de appends diferente
	local continua_ID_years "2017 2018" // años en los que se continua haciendo el append.
	local final_ID_year "2018"   // año final en el cual se va a hacer la búsqueda de los casos para marcar los archivos.

	** Años para buscar los servicios
	local anos_serv "2016 2017 2018"
	local start_serv_year "2016"   // primer año necesita una estructura de appends diferente
```

```
	local continua_serv_years "2017 2018" // años en los que se continua haciendo el append.
	local final_serv_year "2018"   // año final en el cual se va a hacer la búsqueda de servicios
para marcar los archivos.



foreach samp of numlist 1(1)1000 {


	** Cargando la BD de todos los pacientes en el periodo que tienen al menos un código de
ca de pulmón
	use "${bases_cap}/ca_ser_`start_serv_year'-`final_serv_year'_inicial.dta", replace

	** Ajustando fecha de servicio
	gen fx_servicio=date(FechaServicio, "YMD")
	format fx_servicio %td
	gen mes_servicio=mofd(fx_servicio)
	format mes_servicio %tm
	gen year_servicio=year(fx_servicio)
	drop FechaServicio

	** Excluyendo pacientes que no consumieron servicios en el último año de seguimiento
(aplicando CRITERIO DE INCLUSIÓN EXPUESTOS# 1)----------------------------------------- INCLUSION#1
		bys PersonaBasicaID: egen max_servicio=max(year_servicio)
		keep if max_servicio==`final_serv_year'  // este código identifica aquellos
pacientes que consiumieron servicios en el ultimo año de seguimiento

	** Identificando todos los servicios con CIE10 de Ca de Pulmón 2)--------------------------------
----------------------------------------------------- INCLUSION#2
		gen cie10_3d=DiagnosticoCD
		recast str3 cie10_3d, force
		gen ca_dx=1 if cie10_3d=="C34" /*| cie10_3d=="C33" | DiagnosticoCD=="D381"*/
		replace ca_dx=0 if ca_dx!=1

	** Identificando CUPS de uso de servicios oncológicos 2)--------------------------------------------
-------------------------------------------------- INCLUSION#2
		gen 	cups_ca=1 if ProcedimientoCD=="320002"
		replace cups_ca=1 if ProcedimientoCD=="320003"
		replace cups_ca=1 if ProcedimientoCD=="320201"
		replace cups_ca=1 if ProcedimientoCD=="322800"
		replace cups_ca=1 if ProcedimientoCD=="323100"
		replace cups_ca=1 if ProcedimientoCD=="324101"
		replace cups_ca=1 if ProcedimientoCD=="324102"
		replace cups_ca=1 if ProcedimientoCD=="324200"
		replace cups_ca=1 if ProcedimientoCD=="324201"
		replace cups_ca=1 if ProcedimientoCD=="324202"
		replace cups_ca=1 if ProcedimientoCD=="324203"
		replace cups_ca=1 if ProcedimientoCD=="324204"
```

Supporting file

```
replace cups_ca=1 if ProcedimientoCD=="325100"
replace cups_ca=1 if ProcedimientoCD=="325101"
replace cups_ca=1 if ProcedimientoCD=="325102"
replace cups_ca=1 if ProcedimientoCD=="325200"
replace cups_ca=1 if ProcedimientoCD=="325201"
replace cups_ca=1 if ProcedimientoCD=="325202"
replace cups_ca=1 if ProcedimientoCD=="325300"
replace cups_ca=1 if ProcedimientoCD=="325301"
replace cups_ca=1 if ProcedimientoCD=="325302"
replace cups_ca=1 if ProcedimientoCD=="326101"
replace cups_ca=1 if ProcedimientoCD=="332203"
replace cups_ca=1 if ProcedimientoCD=="332204"
replace cups_ca=1 if ProcedimientoCD=="332206"
replace cups_ca=1 if ProcedimientoCD=="332207"
replace cups_ca=1 if ProcedimientoCD=="332401"
replace cups_ca=1 if ProcedimientoCD=="332500"
replace cups_ca=1 if ProcedimientoCD=="332501"
replace cups_ca=1 if ProcedimientoCD=="332601"
replace cups_ca=1 if ProcedimientoCD=="332701"
replace cups_ca=1 if ProcedimientoCD=="332702"
replace cups_ca=1 if ProcedimientoCD=="332703"
replace cups_ca=1 if ProcedimientoCD=="332704"
replace cups_ca=1 if ProcedimientoCD=="332801"
replace cups_ca=1 if ProcedimientoCD=="340200"
replace cups_ca=1 if ProcedimientoCD=="340201"
replace cups_ca=1 if ProcedimientoCD=="342100"
replace cups_ca=1 if ProcedimientoCD=="320001"
replace cups_ca=1 if ProcedimientoCD=="320202"
replace cups_ca=1 if ProcedimientoCD=="334400"
replace cups_ca=1 if ProcedimientoCD=="549004"
replace cups_ca=1 if ProcedimientoCD=="549011"
replace cups_ca=1 if ProcedimientoCD=="992501"
replace cups_ca=1 if ProcedimientoCD=="992502"
replace cups_ca=1 if ProcedimientoCD=="992503"
replace cups_ca=1 if ProcedimientoCD=="992504"
replace cups_ca=1 if ProcedimientoCD=="992505"
replace cups_ca=1 if ProcedimientoCD=="992510"
replace cups_ca=1 if ProcedimientoCD=="992506"
replace cups_ca=1 if ProcedimientoCD=="992507"
replace cups_ca=1 if ProcedimientoCD=="890287"
replace cups_ca=1 if ProcedimientoCD=="890387"
replace cups_ca=1 if ProcedimientoCD=="890487"
replace cups_ca=1 if ProcedimientoCD=="922506"
replace cups_ca=1 if ProcedimientoCD=="922800"
replace cups_ca=1 if ProcedimientoCD=="922801"
replace cups_ca=1 if ProcedimientoCD=="S22222"
replace cups_ca=0 if cups_ca!=1
```

Supporting file

```
** Identificando características de base

bys PersonaBasicaID: egen residencia=mode(MunicipioCD) if  year_servicio==`final_serv_year' |
year_servicio==(`final_serv_year'-1), maxmode // ambitosprocedimientocd=="A" &
bys PersonaBasicaID: egen eps=mode(CodigoAdministradora) if year_servicio==`final_serv_year' |
year_servicio==(`final_serv_year'-1), maxmode

gen f_nac=date(AnnoNacimiento, "YMD")
replace f_nac=date(AnnoNacimiento,"Y") if f_nac==.
format f_nac %td
drop AnnoNacimiento

bys PersonaBasicaID: egen sexo=mode(Sexo), maxmode

collapse (max) f_nac residencia (first) eps sexo (max) ca_dx cups_ca, by(PersonaBasicaID
mes_servicio)

collapse (max) f_nac residencia (first) eps sexo  (sum) ca_dx cups_ca, by(PersonaBasicaID)



***///////////////////////////////////////////////////////////////////////////////////////////////
////
*****************************************************************************
*************************
*** Linea para hacer el bootstraping

bsample, cluster(PersonaBasicaID) idcluster(PersonaBasicaID_s)

*****************************************************************************
**************************
***///////////////////////////////////////////////////////////////////////////////////////////////
////



** Sexo
gen mujer=1 if sexo=="F"
replace mujer=0 if sexo=="M"

drop if mujer==.

** Depto/Region

gen depto=int(residencia/1000)
```

Supporting file

```
label define depto 5 "Antioquia" 8 "Atlántico" 11 "Bogotá" 13 "Bolívar" 15 "Boyacá" 17 "Caldas" 18
"Caquetá" /*
*/ 19 "Cauca" 20 "Cesar" 23 "Córdoba" 25 "Cundinamarca" 27 "Choco" 41 "Huila" 44 "La Guajira"
47 "Magdalena" /*
*/ 50 "Meta" 52 "Nariño" 54 "N de Santander" 63 "Quindío" 66 "Risaralda" 68 "Santander" 70
"Sucre" 73 "Tolima" /*
*/ 76 "Valle del Cauca" 81 "Arauca" 85 "Casanare" 86 "Putumayo" 88 "San Andrés" 91 "Amazonas"
94 "Guainía" 95 "Guaviare" 97 "Vaupés" 99 "Vichada"

label values depto depto

gen region=1 if depto==8 | depto==13 | depto==20 | depto==23 | depto==44 | depto==47 |
depto==70 | depto==88
replace region=2 if depto==11
replace region=3 if depto==5 | depto==17 | depto==18 | depto==41 | depto==63 | depto==66 |
depto==73
replace region=4 if depto==15 | depto==25 | depto==50 | depto==54 | depto==68
replace region=5 if depto==91 | depto==81 | depto==85 | depto==86 | depto==88 | depto==94 |
depto==95 | depto==97 | depto==99
replace region=6 if depto==76 | depto==19 | depto==27 | depto==52

label define region 1 "Atlántica" 2 "Bogotá DC" 3 "Central" 4 "Oriental" 5 "Orinoquía - Amazonía" 6
"Pacífica"
label values region region


*** Definiendo grupos de edad ***

gen age=(21185-f_nac)/365.25   // fecha 21185 1 de enero de 2018
keep if age>=20 & age<=110
gen age_group = 1 if age<45
replace age_group = 2 if age>=45 & age<65
replace age_group = 3 if age>=65

label define age_group 1 "20 a 44" 2 "45 a 64" 3 "65 o más"
label values age_group age_group

**********************************************************************


** Generar las definiciones

gen def_sen=1 if ca_dx>=4
gen def_esp=1 if ca_dx>=4 & cups_ca!=0


** Generando la base de datos
```

```
collapse (sum) def_sen def_esp, by(mujer region age_group)


save
"${resultados_cap}/bootstrapping/boots_Contributivo_n_3yrs_`final_serv_year'_rep`samp'.dta",
replace

}



********************************************************************************
******
**# Prevalencia por SEXO
********************************************************************************
*******

foreach samp of numlist 1(1)1000 {

* Cargando la base
use "${resultados_cap}/bootstrapping/boots_Contributivo_n_3yrs_2018_rep`samp'.dta", clear

********************************************************************************
*******
** Estimando la prevalencia por sexo y grupo etario

merge 1:1 mujer region age_group using
"/home/javier/Documentos/Ca_pulmon/Bases_cap/Denominadores_BDUA.dta"
drop _merge

collapse (sum) def_sen def_esp denominador_2018, by(mujer)

gen cum_deno= sum(denominador_2018)
egen tot_denominador_2018 = max(cum_deno)
drop cum_deno

gen cumsen= sum(def_sen)
gen cumesp= sum(def_esp)

egen tot_sen = max(cumsen)
egen tot_esp = max(cumesp)

drop cumsen cumesp

gen p_str_sen= (def_sen/denominador_2018)*100000
gen p_str_esp= (def_esp/denominador_2018)*100000

gen p_total_sen = (tot_sen/tot_denominador_2018)*100000
```

Supporting file

```
gen p_total_esp = (tot_esp/tot_denominador_2018)*100000


save "${resultados_cap}/bootstrapping/Pre_estimates_sex_2018_rep`samp'.dta", replace

}


** Uniendo las estimaciones del bootstraping para poder sacar las medidas agregadas:


use "${resultados_cap}/bootstrapping/Pre_estimates_sex_2018_rep1.dta", clear

foreach samp of numlist 2(1)1000 {

append using "${resultados_cap}/bootstrapping/Pre_estimates_sex_2018_rep`samp'.dta"

}

** Haciendo las estimaciones de las prevalencias específicas y sensibles por estratos (sexo y edad)

table ( mujer) (), statistic(mean p_str_sen )  statistic(p25 p_str_sen) statistic(p75 p_str_sen)
statistic(mean p_str_esp )  statistic(p25 p_str_esp) statistic(p75 p_str_esp) nototals
collect label levels mujer 0 "Hombre" 1 "Mujer", modify
collect export "${resultados_cap}/Prevalencias_estrato_sexo_2018.docx", as(docx) replace

********************************************************************************
******
**# Prevalencia por GRUPO ETARIO
********************************************************************************
*******

foreach samp of numlist 1(1)1000 {

* Cargando la base
use "${resultados_cap}/bootstrapping/boots_Contributivo_n_3yrs_2018_rep`samp'.dta", clear

********************************************************************************
*******
** Estimando la prevalencia por sexo y grupo etario

merge 1:1 mujer region age_group using
"/home/javier/Documentos/Ca_pulmon/Bases_cap/Denominadores_BDUA.dta"
drop _merge

collapse (sum) def_sen def_esp denominador_2018, by(age_group)

gen cum_deno= sum(denominador_2018)
```

```
egen tot_denominador_2018 = max(cum_deno)
drop cum_deno

gen cumsen= sum(def_sen)
gen cumesp= sum(def_esp)

egen tot_sen = max(cumsen)
egen tot_esp = max(cumesp)

drop cumsen cumesp

gen p_str_sen= (def_sen/denominador_2018)*100000
gen p_str_esp= (def_esp/denominador_2018)*100000

gen p_total_sen = (tot_sen/tot_denominador_2018)*100000
gen p_total_esp = (tot_esp/tot_denominador_2018)*100000


save "${resultados_cap}/bootstrapping/Pre_estimates_age_2018_rep`samp'.dta", replace

}


** Uniendo las estimaciones del bootstraping para poder sacar las medidas agregadas:


use "${resultados_cap}/bootstrapping/Pre_estimates_age_2018_rep1.dta", clear

foreach samp of numlist 2(1)1000 {

append using "${resultados_cap}/bootstrapping/Pre_estimates_age_2018_rep`samp'.dta"

}

** Haciendo las estimaciones de las prevalencias específicas y sensibles por estratos (sexo y edad)

table (age_group) (), statistic(mean p_str_sen )  statistic(p25 p_str_sen) statistic(p75 p_str_sen)
statistic(mean p_str_esp )  statistic(p25 p_str_esp) statistic(p75 p_str_esp) nototals
collect export "${resultados_cap}/Prevalencias_estrato_edad_2018.docx", as(docx) replace

*********************************************************************************
******
**# Prevalencia por REGION
*********************************************************************************
*******

foreach samp of numlist 1(1)1000 {
```

Supporting file

```
* Cargando la base
use
"${resultados_cap}/bootstrapping/boots_Contributivo_n_3yrs_`final_serv_year'_rep`samp'.dta",
clear


********************************************************************************
*******
** Estimando la prevalencia por sexo y grupo etario

merge 1:1 mujer region age_group using
"/home/javier/Documentos/Ca_pulmon/Bases_cap/Denominadores_BDUA.dta"
drop _merge

collapse (sum) def_sen def_esp denominador_`final_serv_year', by(region)

gen cum_deno= sum(denominador_`final_serv_year')
egen tot_denominador_`final_serv_year' = max(cum_deno)
drop cum_deno

gen cumsen= sum(def_sen)
gen cumesp= sum(def_esp)

egen tot_sen = max(cumsen)
egen tot_esp = max(cumesp)

drop cumsen cumesp

gen p_str_sen= (def_sen/denominador_`final_serv_year')*100000
gen p_str_esp= (def_esp/denominador_`final_serv_year')*100000

gen p_total_sen = (tot_sen/tot_denominador_`final_serv_year')*100000
gen p_total_esp = (tot_esp/tot_denominador_`final_serv_year')*100000


save "${resultados_cap}/bootstrapping/Pre_estimates_region_`final_serv_year'_rep`samp'.dta",
replace

}


** Uniendo las estimaciones del bootstraping para poder sacar las medidas agregadas:


use "${resultados_cap}/bootstrapping/Pre_estimates_region_`final_serv_year'_rep1.dta", clear

foreach samp of numlist 2(1)1000 {
```

append using
"${resultados_cap}/bootstrapping/Pre_estimates_region_`final_serv_year'_rep`samp'.dta"

}

** Haciendo las estimaciones de las prevalencias específicas y sensibles por estratos (REGION)

table (region) (), statistic(mean p_str_sen) statistic(p25 p_str_sen) statistic(p75 p_str_sen)
statistic(mean p_str_esp) statistic(p25 p_str_esp) statistic(p75 p_str_esp) nototals
collect export "${resultados_cap}/Prevalencias_estrato_region_`final_serv_year'.docx", as(docx)
replace

** Haciendo la tabla de prevalencia general
table () (), statistic(mean p_str_sen) statistic(p25 p_str_sen) statistic(p75 p_str_sen)
statistic(mean p_str_esp) statistic(p25 p_str_esp) statistic(p75 p_str_esp) nototals
collect export "${resultados_cap}/Prevalencias_total_`final_serv_year'.docx", as(docx) replace

****
log close

****

6_Prevalence_estimation_cap_2019_contributory

****

* Opening the log file
	clear all
	*Log_files
	global logs_cap /home/javier/Documentos/Ca_pulmon/1_log_files
	*Ruta bases ORIGINALES:
	global bases_ori /home/javier/Documentos/BASES_ORI
	*Ruta bases CS:
	global bases_cap /home/javier/Documentos/Ca_pulmon/Bases_cap
	*Ruta resultados
	global resultados_cap /home/javier/Documentos/Ca_pulmon/3_Resultados/Contributivo
	*Abriendo el log
	log using "${logs_cap}/Prevalence_estimation_2019-contributivo_bootstrapping.smcl",
replace

****
****
**# Preparación de las bases para el bootstrapping

** Estableciendo los parametros para correr la identificación de sujetos con CAP

Supporting file

```
	** Años para buscar los ID en la UPC
	local anos_ID "2017 2018 2019"
	local start_ID_year "2017"   // primer año necesita una estructura de appends diferente
	local continua_ID_years "2018 2019" // años en los que se continua haciendo el append.
	local final_ID_year "2019"   // año final en el cual se va a hacer la búsqueda de los casos 
para marcar los archivos.


	** Años para buscar los servicios
	local anos_serv "2017 2018 2019"
	local start_serv_year "2017"   // primer año necesita una estructura de appends diferente
	local continua_serv_years "2018 2019" // años en los que se continua haciendo el append.
	local final_serv_year "2019"   // año final en el cual se va a hacer la búsqueda de servicios 
para marcar los archivos.



foreach samp of numlist 1(1)1000 {


	** Cargando la BD de todos los pacientes en el periodo que tienen al menos un código de 
ca de pulmón
	use "${bases_cap}/ca_ser_`start_serv_year'-`final_serv_year'_inicial.dta", replace

	** Ajustando fecha de servicio
	gen fx_servicio=date(FechaServicio, "YMD")
	format fx_servicio %td
	gen mes_servicio=mofd(fx_servicio)
	format mes_servicio %tm
	gen year_servicio=year(fx_servicio)
	drop FechaServicio

	** Excluyendo pacientes que no consumieron servicios en el último año de seguimiento 
(aplicando CRITERIO DE INCLUSIÓN EXPUESTOS# 1)---------------------------------------- INCLUSION#1
		bys PersonaBasicaID: egen max_servicio=max(year_servicio)
		keep if max_servicio==`final_serv_year'  // este código identifica aquellos 
pacientes que consiumieron servicios en el ultimo año de seguimiento

	** Identificando todos los servicios con CIE10 de Ca de Pulmón 2)--------------------------------
--------------------------------------------------- INCLUSION#2
		gen cie10_3d=DiagnosticoCD
		recast str3 cie10_3d, force
		gen ca_dx=1 if cie10_3d=="C34" /*| cie10_3d=="C33" | DiagnosticoCD=="D381"*/
		replace ca_dx=0 if ca_dx!=1

	** Identificando CUPS de uso de servicios oncológicos 2)-------------------------------------------
----------------------------------------------- INCLUSION#2
```

Supporting file

```
gen       cups_ca=1 if ProcedimientoCD=="320002"
replace cups_ca=1 if ProcedimientoCD=="320003"
replace cups_ca=1 if ProcedimientoCD=="320201"
replace cups_ca=1 if ProcedimientoCD=="322800"
replace cups_ca=1 if ProcedimientoCD=="323100"
replace cups_ca=1 if ProcedimientoCD=="324101"
replace cups_ca=1 if ProcedimientoCD=="324102"
replace cups_ca=1 if ProcedimientoCD=="324200"
replace cups_ca=1 if ProcedimientoCD=="324201"
replace cups_ca=1 if ProcedimientoCD=="324202"
replace cups_ca=1 if ProcedimientoCD=="324203"
replace cups_ca=1 if ProcedimientoCD=="324204"
replace cups_ca=1 if ProcedimientoCD=="325100"
replace cups_ca=1 if ProcedimientoCD=="325101"
replace cups_ca=1 if ProcedimientoCD=="325102"
replace cups_ca=1 if ProcedimientoCD=="325200"
replace cups_ca=1 if ProcedimientoCD=="325201"
replace cups_ca=1 if ProcedimientoCD=="325202"
replace cups_ca=1 if ProcedimientoCD=="325300"
replace cups_ca=1 if ProcedimientoCD=="325301"
replace cups_ca=1 if ProcedimientoCD=="325302"
replace cups_ca=1 if ProcedimientoCD=="326101"
replace cups_ca=1 if ProcedimientoCD=="332203"
replace cups_ca=1 if ProcedimientoCD=="332204"
replace cups_ca=1 if ProcedimientoCD=="332206"
replace cups_ca=1 if ProcedimientoCD=="332207"
replace cups_ca=1 if ProcedimientoCD=="332401"
replace cups_ca=1 if ProcedimientoCD=="332500"
replace cups_ca=1 if ProcedimientoCD=="332501"
replace cups_ca=1 if ProcedimientoCD=="332601"
replace cups_ca=1 if ProcedimientoCD=="332701"
replace cups_ca=1 if ProcedimientoCD=="332702"
replace cups_ca=1 if ProcedimientoCD=="332703"
replace cups_ca=1 if ProcedimientoCD=="332704"
replace cups_ca=1 if ProcedimientoCD=="332801"
replace cups_ca=1 if ProcedimientoCD=="340200"
replace cups_ca=1 if ProcedimientoCD=="340201"
replace cups_ca=1 if ProcedimientoCD=="342100"
replace cups_ca=1 if ProcedimientoCD=="320001"
replace cups_ca=1 if ProcedimientoCD=="320202"
replace cups_ca=1 if ProcedimientoCD=="334400"
replace cups_ca=1 if ProcedimientoCD=="549004"
replace cups_ca=1 if ProcedimientoCD=="549011"
replace cups_ca=1 if ProcedimientoCD=="992501"
replace cups_ca=1 if ProcedimientoCD=="992502"
replace cups_ca=1 if ProcedimientoCD=="992503"
replace cups_ca=1 if ProcedimientoCD=="992504"
replace cups_ca=1 if ProcedimientoCD=="992505"
```

```
replace cups_ca=1 if ProcedimientoCD=="992510"
replace cups_ca=1 if ProcedimientoCD=="992506"
replace cups_ca=1 if ProcedimientoCD=="992507"
replace cups_ca=1 if ProcedimientoCD=="890287"
replace cups_ca=1 if ProcedimientoCD=="890387"
replace cups_ca=1 if ProcedimientoCD=="890487"
replace cups_ca=1 if ProcedimientoCD=="922506"
replace cups_ca=1 if ProcedimientoCD=="922800"
replace cups_ca=1 if ProcedimientoCD=="922801"
replace cups_ca=1 if ProcedimientoCD=="S22222"
replace cups_ca=0 if cups_ca!=1

** Identificando características de base

bys PersonaBasicaID: egen residencia=mode(MunicipioCD) if  year_servicio==`final_serv_year' | year_servicio==(`final_serv_year'-1), maxmode // ambitosprocedimientocd=="A" &
bys PersonaBasicaID: egen eps=mode(CodigoAdministradora) if year_servicio==`final_serv_year' | year_servicio==(`final_serv_year'-1), maxmode

gen f_nac=date(AnnoNacimiento, "YMD")
replace f_nac=date(AnnoNacimiento,"Y") if f_nac==.
format f_nac %td
drop AnnoNacimiento

bys PersonaBasicaID: egen sexo=mode(Sexo), maxmode

collapse (max) f_nac residencia (first) eps sexo (max) ca_dx cups_ca, by(PersonaBasicaID mes_servicio)

collapse (max) f_nac residencia (first) eps sexo  (sum) ca_dx cups_ca, by(PersonaBasicaID)



***//////////////////////////////////////////////////////////////////////////////////////////////
////
*****************************************************************************
*************************
*** Linea para hacer el bootstraping

bsample, cluster(PersonaBasicaID) idcluster(PersonaBasicaID_s)

*****************************************************************************
**************************
***//////////////////////////////////////////////////////////////////////////////////////////////
////
```

Supporting file

```
** Sexo
gen mujer=1 if sexo=="F"
replace mujer=0 if sexo=="M"

drop if mujer==.

** Depto/Region

gen depto=int(residencia/1000)

label define depto 5 "Antioquia" 8 "Atlántico" 11 "Bogotá" 13 "Bolívar" 15 "Boyacá" 17 "Caldas" 18
"Caquetá" /*
*/ 19 "Cauca" 20 "Cesar" 23 "Córdoba" 25 "Cundinamarca" 27 "Choco" 41 "Huila" 44 "La Guajira"
47 "Magdalena" /*
*/ 50 "Meta" 52 "Nariño" 54 "N de Santander" 63 "Quindío" 66 "Risaralda" 68 "Santander" 70
"Sucre" 73 "Tolima" /*
*/ 76 "Valle del Cauca" 81 "Arauca" 85 "Casanare" 86 "Putumayo" 88 "San Andrés" 91 "Amazonas"
94 "Guainía" 95 "Guaviare" 97 "Vaupés" 99 "Vichada"

label values depto depto

gen region=1 if depto==8 | depto==13 | depto==20 | depto==23 | depto==44 | depto==47 |
depto==70 | depto==88
replace region=2 if depto==11
replace region=3 if depto==5 | depto==17 | depto==18 | depto==41 | depto==63 | depto==66 |
depto==73
replace region=4 if depto==15 | depto==25 | depto==50 | depto==54 | depto==68
replace region=5 if depto==91 | depto==81 | depto==85 | depto==86 | depto==88 | depto==94 |
depto==95 | depto==97 | depto==99
replace region=6 if depto==76 | depto==19 | depto==27 | depto==52

label define region 1 "Atlántica" 2 "Bogotá DC" 3 "Central" 4 "Oriental" 5 "Orinoquía - Amazonía" 6
"Pacífica"
label values region region


*** Definiendo grupos de edad ***

gen age=(21550-f_nac)/365.25   // fecha 21550 1 de enero de 2019
keep if age>=20 & age<=110
gen age_group = 1 if age<45
replace age_group = 2 if age>=45 & age<65
replace age_group = 3 if age>=65

label define age_group 1 "20 a 44" 2 "45 a 64" 3 "65 o más"
label values age_group age_group
```

Supporting file

```
************************************************************************

** Generar las definiciones

gen def_sen=1 if ca_dx>=4
gen def_esp=1 if ca_dx>=4 & cups_ca!=0


** Generando la base de datos

collapse (sum) def_sen def_esp, by(mujer region age_group)


save
"${resultados_cap}/bootstrapping/boots_Contributivo_n_3yrs_`final_serv_year'_rep`samp'.dta",
replace

}


******************************************************************************
******
**# Prevalencia por SEXO
******************************************************************************
*******

foreach samp of numlist 1(1)1000 {

* Cargando la base
use "${resultados_cap}/bootstrapping/boots_Contributivo_n_3yrs_2019_rep`samp'.dta", clear

******************************************************************************
*******
** Estimando la prevalencia por sexo y grupo etario

merge 1:1 mujer region age_group using
"/home/javier/Documentos/Ca_pulmon/Bases_cap/Denominadores_BDUA.dta"
drop _merge

collapse (sum) def_sen def_esp denominador_2019, by(mujer)

gen cum_deno= sum(denominador_2019)
egen tot_denominador_2019 = max(cum_deno)
drop cum_deno

gen cumsen= sum(def_sen)
```

```
gen cumesp= sum(def_esp)

egen tot_sen = max(cumsen)
egen tot_esp = max(cumesp)

drop cumsen cumesp

gen p_str_sen= (def_sen/denominador_2019)*100000
gen p_str_esp= (def_esp/denominador_2019)*100000

gen p_total_sen = (tot_sen/tot_denominador_2019)*100000
gen p_total_esp = (tot_esp/tot_denominador_2019)*100000


save "${resultados_cap}/bootstrapping/Pre_estimates_sex_2019_rep`samp'.dta", replace

}


** Uniendo las estimaciones del bootstraping para poder sacar las medidas agregadas:


use "${resultados_cap}/bootstrapping/Pre_estimates_sex_2019_rep1.dta", clear

foreach samp of numlist 2(1)1000 {

append using "${resultados_cap}/bootstrapping/Pre_estimates_sex_2019_rep`samp'.dta"

}

** Haciendo las estimaciones de las prevalencias específicas y sensibles por estratos (sexo y edad)

table ( mujer) (), statistic(mean p_str_sen )  statistic(p25 p_str_sen) statistic(p75 p_str_sen)
statistic(mean p_str_esp )  statistic(p25 p_str_esp) statistic(p75 p_str_esp) nototals
collect label levels mujer 0 "Hombre" 1 "Mujer", modify
collect export "${resultados_cap}/Prevalencias_estrato_sexo_2019.docx", as(docx) replace

********************************************************************************
******
**# Prevalencia por GRUPO ETARIO
********************************************************************************
*******

foreach samp of numlist 1(1)1000 {

* Cargando la base
use "${resultados_cap}/bootstrapping/boots_Contributivo_n_3yrs_2019_rep`samp'.dta", clear
```

```
********************************************************************************
*******
** Estimando la prevalencia por sexo y grupo etario

merge 1:1 mujer region age_group using
"/home/javier/Documentos/Ca_pulmon/Bases_cap/Denominadores_BDUA.dta"
drop _merge

collapse (sum) def_sen def_esp denominador_2019, by(age_group)

gen cum_deno= sum(denominador_2019)
egen tot_denominador_2019 = max(cum_deno)
drop cum_deno

gen cumsen= sum(def_sen)
gen cumesp= sum(def_esp)

egen tot_sen = max(cumsen)
egen tot_esp = max(cumesp)

drop cumsen cumesp

gen p_str_sen= (def_sen/denominador_2019)*100000
gen p_str_esp= (def_esp/denominador_2019)*100000

gen p_total_sen = (tot_sen/tot_denominador_2019)*100000
gen p_total_esp = (tot_esp/tot_denominador_2019)*100000


save "${resultados_cap}/bootstrapping/Pre_estimates_age_2019_rep`samp'.dta", replace

}


** Uniendo las estimaciones del bootstraping para poder sacar las medidas agregadas:


use "${resultados_cap}/bootstrapping/Pre_estimates_age_2019_rep1.dta", clear

foreach samp of numlist 2(1)1000 {

append using "${resultados_cap}/bootstrapping/Pre_estimates_age_2019_rep`samp'.dta"

}

** Haciendo las estimaciones de las prevalencias específicas y sensibles por estratos (sexo y edad)
```

```
table (age_group) (), statistic(mean p_str_sen )  statistic(p25 p_str_sen) statistic(p75 p_str_sen)
statistic(mean p_str_esp )  statistic(p25 p_str_esp) statistic(p75 p_str_esp) nototals
collect export "${resultados_cap}/Prevalencias_estrato_edad_2019.docx", as(docx) replace

******************************************************************************
******
**# Prevalencia por REGION
******************************************************************************
*******

foreach samp of numlist 1(1)1000 {

* Cargando la base
use
"${resultados_cap}/bootstrapping/boots_Contributivo_n_3yrs_`final_serv_year'_rep`samp'.dta",
clear

******************************************************************************
*******
** Estimando la prevalencia por sexo y grupo etario

merge 1:1 mujer region age_group using
"/home/javier/Documentos/Ca_pulmon/Bases_cap/Denominadores_BDUA.dta"
drop _merge

collapse (sum) def_sen def_esp denominador_`final_serv_year', by(region)

gen cum_deno= sum(denominador_`final_serv_year')
egen tot_denominador_`final_serv_year' = max(cum_deno)
drop cum_deno

gen cumsen= sum(def_sen)
gen cumesp= sum(def_esp)

egen tot_sen = max(cumsen)
egen tot_esp = max(cumesp)

drop cumsen cumesp

gen p_str_sen= (def_sen/denominador_`final_serv_year')*100000
gen p_str_esp= (def_esp/denominador_`final_serv_year')*100000

gen p_total_sen = (tot_sen/tot_denominador_`final_serv_year')*100000
gen p_total_esp = (tot_esp/tot_denominador_`final_serv_year')*100000


save "${resultados_cap}/bootstrapping/Pre_estimates_region_`final_serv_year'_rep`samp'.dta",
replace
```

```
}

** Uniendo las estimaciones del bootstraping para poder sacar las medidas agregadas:

use "${resultados_cap}/bootstrapping/Pre_estimates_region_`final_serv_year'_rep1.dta", clear

foreach samp of numlist 2(1)1000 {

append using
"${resultados_cap}/bootstrapping/Pre_estimates_region_`final_serv_year'_rep`samp'.dta"

}

** Haciendo las estimaciones de las prevalencias específicas y sensibles por estratos (REGION)

table (region) (), statistic(mean p_str_sen )  statistic(p25 p_str_sen) statistic(p75 p_str_sen)
statistic(mean p_str_esp )  statistic(p25 p_str_esp) statistic(p75 p_str_esp) nototals
collect export "${resultados_cap}/Prevalencias_estrato_region_`final_serv_year'.docx", as(docx)
replace

** Haciendo la tabla de prevalencia general
table () (), statistic(mean p_str_sen )  statistic(p25 p_str_sen) statistic(p75 p_str_sen)
statistic(mean p_str_esp )  statistic(p25 p_str_esp) statistic(p75 p_str_esp) nototals
collect export "${resultados_cap}/Prevalencias_total_`final_serv_year'.docx", as(docx) replace

log close
```

Data analysis – Subsidized Regime – phase 2/Prevalence estimation

****
4_Prevalence_estimation_cap_2017_subsidized
****

Supporting file

```
* Opening the log file
	clear all
	*Log_files
	global logs_cap /home/javier/Documentos/Ca_pulmon/1_log_files
	*Ruta bases ORIGINALES:
	global bases_ori /home/javier/Documentos/BASES_ORI
	*Ruta bases CS:
	global bases_cap /home/javier/Documentos/Ca_pulmon/Bases_cap
	*Ruta resultados
	global resultados_cap /home/javier/Documentos/Ca_pulmon/3_Resultados/Contributivo
	*Abriendo el log
	log using "${logs_cap}/Prevalence_estimation_2017-contributivo_bootstrapping.smcl",
replace

****************************************************************************
*****************************
**# Preparación de las bases para el bootstrapping



** Estableciendo los parametros para correr la identificación de sujetos con CAP

	** Años para buscar los ID en la UPC
	local anos_ID "2015 2016 2017"
	local start_ID_year "2015"   // primer año necesita una estructura de appends diferente
	local continua_ID_years "2016 2017" // años en los que se continua haciendo el append.
	local final_ID_year "2017"   // año final en el cual se va a hacer la búsqueda de los casos
para marcar los archivos.


	** Años para buscar los servicios
	local anos_serv "2015 2016 2017"
	local start_serv_year "2015"   // primer año necesita una estructura de appends diferente
	local continua_serv_years "2016 2017" // años en los que se continua haciendo el append.
	local final_serv_year "2017"   // año final en el cual se va a hacer la búsqueda de servicios
para marcar los archivos.



foreach samp of numlist 1(1)1000 {


	** Cargando la BD de todos los pacientes en el periodo que tienen al menos un código de
ca de pulmón
	use "${bases_cap}/ca_ser_`start_serv_year'-`final_serv_year'_inicial.dta", replace

	** Ajustando fecha de servicio
	gen fx_servicio=date(FechaServicio, "YMD")
```

Supporting file

```
format fx_servicio %td
gen mes_servicio=mofd(fx_servicio)
format mes_servicio %tm
gen year_servicio=year(fx_servicio)
drop FechaServicio

** Excluyendo pacientes que no consumieron servicios en el 2017 (aplicando CRITERIO DE INCLUSIÓN EXPUESTOS# 1)---------------------------------------- INCLUSION#1
	bys PersonaBasicaID: egen max_servicio=max(year_servicio)
	keep if max_servicio==`final_serv_year'  // este código identifica aquellos pacientes que consiumieron servicios en el año 2017
	//** 3,455,036 observations deleted
	//** quedan 6,205,101 observaciones

	**drop if year_servicio == 2017 // con este código eliminamos de la estimación de casos prevalentes de 3 años (2014,2015 y 2016) las observaciones de servicios correspondientes al año 2017
	//drop if year_servicio == 2014

** Identificando todos los servicios con CIE10 de Ca de Pulmón 2)----------------------------------------------------------------------------------- INCLUSION#2
	gen cie10_3d=DiagnosticoCD
	recast str3 cie10_3d, force
	gen ca_dx=1 if cie10_3d=="C34" /*| cie10_3d=="C33" | DiagnosticoCD=="D381"*/
	replace ca_dx=0 if ca_dx!=1

** Identificando CUPS de uso de servicios oncológicos 2)------------------------------------------------------------------------------------------- INCLUSION#2
	gen	cups_ca=1 if ProcedimientoCD=="320002"
	replace cups_ca=1 if ProcedimientoCD=="320003"
	replace cups_ca=1 if ProcedimientoCD=="320201"
	replace cups_ca=1 if ProcedimientoCD=="322800"
	replace cups_ca=1 if ProcedimientoCD=="323100"
	replace cups_ca=1 if ProcedimientoCD=="324101"
	replace cups_ca=1 if ProcedimientoCD=="324102"
	replace cups_ca=1 if ProcedimientoCD=="324200"
	replace cups_ca=1 if ProcedimientoCD=="324201"
	replace cups_ca=1 if ProcedimientoCD=="324202"
	replace cups_ca=1 if ProcedimientoCD=="324203"
	replace cups_ca=1 if ProcedimientoCD=="324204"
	replace cups_ca=1 if ProcedimientoCD=="325100"
	replace cups_ca=1 if ProcedimientoCD=="325101"
	replace cups_ca=1 if ProcedimientoCD=="325102"
	replace cups_ca=1 if ProcedimientoCD=="325200"
	replace cups_ca=1 if ProcedimientoCD=="325201"
	replace cups_ca=1 if ProcedimientoCD=="325202"
	replace cups_ca=1 if ProcedimientoCD=="325300"
	replace cups_ca=1 if ProcedimientoCD=="325301"
```

```
replace cups_ca=1 if ProcedimientoCD=="325302"
replace cups_ca=1 if ProcedimientoCD=="326101"
replace cups_ca=1 if ProcedimientoCD=="332203"
replace cups_ca=1 if ProcedimientoCD=="332204"
replace cups_ca=1 if ProcedimientoCD=="332206"
replace cups_ca=1 if ProcedimientoCD=="332207"
replace cups_ca=1 if ProcedimientoCD=="332401"
replace cups_ca=1 if ProcedimientoCD=="332500"
replace cups_ca=1 if ProcedimientoCD=="332501"
replace cups_ca=1 if ProcedimientoCD=="332601"
replace cups_ca=1 if ProcedimientoCD=="332701"
replace cups_ca=1 if ProcedimientoCD=="332702"
replace cups_ca=1 if ProcedimientoCD=="332703"
replace cups_ca=1 if ProcedimientoCD=="332704"
replace cups_ca=1 if ProcedimientoCD=="332801"
replace cups_ca=1 if ProcedimientoCD=="340200"
replace cups_ca=1 if ProcedimientoCD=="340201"
replace cups_ca=1 if ProcedimientoCD=="342100"
replace cups_ca=1 if ProcedimientoCD=="320001"
replace cups_ca=1 if ProcedimientoCD=="320202"
replace cups_ca=1 if ProcedimientoCD=="334400"
replace cups_ca=1 if ProcedimientoCD=="549004"
replace cups_ca=1 if ProcedimientoCD=="549011"
replace cups_ca=1 if ProcedimientoCD=="992501"
replace cups_ca=1 if ProcedimientoCD=="992502"
replace cups_ca=1 if ProcedimientoCD=="992503"
replace cups_ca=1 if ProcedimientoCD=="992504"
replace cups_ca=1 if ProcedimientoCD=="992505"
replace cups_ca=1 if ProcedimientoCD=="992510"
replace cups_ca=1 if ProcedimientoCD=="992506"
replace cups_ca=1 if ProcedimientoCD=="992507"
replace cups_ca=1 if ProcedimientoCD=="890287"
replace cups_ca=1 if ProcedimientoCD=="890387"
replace cups_ca=1 if ProcedimientoCD=="890487"
replace cups_ca=1 if ProcedimientoCD=="922506"
replace cups_ca=1 if ProcedimientoCD=="922800"
replace cups_ca=1 if ProcedimientoCD=="922801"
replace cups_ca=1 if ProcedimientoCD=="S22222"
replace cups_ca=0 if cups_ca!=1

** Identificando características de base

bys PersonaBasicaID: egen residencia=mode(MunicipioCD) if  year_servicio==`final_serv_year' | year_servicio==(`final_serv_year'-1), maxmode // ambitosprocedimientocd=="A" &
bys PersonaBasicaID: egen eps=mode(CodigoAdministradora) if year_servicio==`final_serv_year' | year_servicio==(`final_serv_year'-1), maxmode

gen f_nac=date(AnnoNacimiento, "YMD")
```

```
replace f_nac=date(AnnoNacimiento,"Y") if f_nac==.
format f_nac %td
drop AnnoNacimiento

bys PersonaBasicaID: egen sexo=mode(Sexo), maxmode

collapse (max) f_nac residencia (first) eps sexo (max) ca_dx cups_ca, by(PersonaBasicaID
mes_servicio)

collapse (max) f_nac residencia (first) eps sexo  (sum) ca_dx cups_ca, by(PersonaBasicaID)




***////////////////////////////////////////////////////////////////////////////////////////////////////////////
////
*****************************************************************************************
*************************
*** Linea para hacer el bootstraping

bsample, cluster(PersonaBasicaID) idcluster(PersonaBasicaID_s)

*****************************************************************************************
**************************
***////////////////////////////////////////////////////////////////////////////////////////////////////////////
////




** Sexo
gen mujer=1 if sexo=="F"
replace mujer=0 if sexo=="M"

drop if mujer==.

** Depto/Region

gen depto=int(residencia/1000)

label define depto 5 "Antioquia" 8 "Atlántico" 11 "Bogotá" 13 "Bolívar" 15 "Boyacá" 17 "Caldas" 18
"Caquetá" /*
*/ 19 "Cauca" 20 "Cesar" 23 "Córdoba" 25 "Cundinamarca" 27 "Choco" 41 "Huila" 44 "La Guajira"
47 "Magdalena" /*
*/ 50 "Meta" 52 "Nariño" 54 "N de Santander" 63 "Quindío" 66 "Risaralda" 68 "Santander" 70
"Sucre" 73 "Tolima" /*
*/ 76 "Valle del Cauca" 81 "Arauca" 85 "Casanare" 86 "Putumayo" 88 "San Andrés" 91 "Amazonas"
94 "Guainía" 95 "Guaviare" 97 "Vaupés" 99 "Vichada"
```

```
label values depto depto

gen region=1 if depto==8 | depto==13 | depto==20 | depto==23 | depto==44 | depto==47 |
depto==70 | depto==88
replace region=2 if depto==11
replace region=3 if depto==5 | depto==17 | depto==18 | depto==41 | depto==63 | depto==66 |
depto==73
replace region=4 if depto==15 | depto==25 | depto==50 | depto==54 | depto==68
replace region=5 if depto==91 | depto==81 | depto==85 | depto==86 | depto==88 | depto==94 |
depto==95 | depto==97 | depto==99
replace region=6 if depto==76 | depto==19 | depto==27 | depto==52

label define region 1 "Atlántica" 2 "Bogotá DC" 3 "Central" 4 "Oriental" 5 "Orinoquía - Amazonía" 6
"Pacífica"
label values region region


*** Definiendo grupos de edad ***

gen age=(`final_serv_year'5-f_nac)/365.25   // fecha 20185 1 de enero de 2018
keep if age>=20 & age<=110
gen age_group = 1 if age<45
replace age_group = 2 if age>=45 & age<65
replace age_group = 3 if age>=65

label define age_group 1 "20 a 44" 2 "45 a 64" 3 "65 o más"
label values age_group age_group

**********************************************************************


** Generar las definiciones

gen def_sen=1 if ca_dx>=4
gen def_esp=1 if ca_dx>=4 & cups_ca!=0


** Generando la base de datos

collapse (sum) def_sen def_esp, by(mujer region age_group)


save
"${resultados_cap}/bootstrapping/boots_Contributivo_n_3yrs_`final_serv_year'_rep`samp'.dta",
replace

}
```

```
********************************************************************************
******
**# Prevalencia por SEXO
********************************************************************************
*******

foreach samp of numlist 1(1)1000 {

* Cargando la base
use "${resultados_cap}/bootstrapping/boots_Contributivo_n_3yrs_2017_rep`samp'.dta", clear

********************************************************************************
*******
** Estimando la prevalencia por sexo y grupo etario

merge 1:1 mujer region age_group using
"/home/javier/Documentos/Ca_pulmon/Bases_cap/Denominadores_BDUA.dta"
drop _merge

collapse (sum) def_sen def_esp denominador_2017, by(mujer)

gen cum_deno= sum(denominador_2017)
egen tot_denominador_2017 = max(cum_deno)
drop cum_deno

gen cumsen= sum(def_sen)
gen cumesp= sum(def_esp)

egen tot_sen = max(cumsen)
egen tot_esp = max(cumesp)

drop cumsen cumesp

gen p_str_sen= (def_sen/denominador_2017)*100000
gen p_str_esp= (def_esp/denominador_2017)*100000

gen p_total_sen = (tot_sen/tot_denominador_2017)*100000
gen p_total_esp = (tot_esp/tot_denominador_2017)*100000


save "${resultados_cap}/bootstrapping/Pre_estimates_sex_2017_rep`samp'.dta", replace

}
```

Supporting file

```
** Uniendo las estimaciones del bootstraping para poder sacar las medidas agregadas:


use "${resultados_cap}/bootstrapping/Pre_estimates_sex_2017_rep1.dta", clear

foreach samp of numlist 2(1)1000 {

append using "${resultados_cap}/bootstrapping/Pre_estimates_sex_2017_rep`samp'.dta"

}

** Haciendo las estimaciones de las prevalencias específicas y sensibles por estratos (sexo y edad)

table ( mujer) (), statistic(mean p_str_sen )  statistic(p25 p_str_sen) statistic(p75 p_str_sen)
statistic(mean p_str_esp )  statistic(p25 p_str_esp) statistic(p75 p_str_esp) nototals
collect label levels mujer 0 "Hombre" 1 "Mujer", modify
collect export "${resultados_cap}/Prevalencias_estrato_sexo_2017.docx", as(docx) replace

*********************************************************************************
******
**# Prevalencia por GRUPO ETARIO
*********************************************************************************
*******

foreach samp of numlist 1(1)1000 {

* Cargando la base
use "${resultados_cap}/bootstrapping/boots_Contributivo_n_3yrs_2017_rep`samp'.dta", clear

*********************************************************************************
*******
** Estimando la prevalencia por sexo y grupo etario

merge 1:1 mujer region age_group using
"/home/javier/Documentos/Ca_pulmon/Bases_cap/Denominadores_BDUA.dta"
drop _merge

collapse (sum) def_sen def_esp denominador_2017, by(age_group)

gen cum_deno= sum(denominador_2017)
egen tot_denominador_2017 = max(cum_deno)
drop cum_deno

gen cumsen= sum(def_sen)
gen cumesp= sum(def_esp)

egen tot_sen = max(cumsen)
```

```
egen tot_esp = max(cumesp)

drop cumsen cumesp

gen p_str_sen= (def_sen/denominador_2017)*100000
gen p_str_esp= (def_esp/denominador_2017)*100000

gen p_total_sen = (tot_sen/tot_denominador_2017)*100000
gen p_total_esp = (tot_esp/tot_denominador_2017)*100000


save "${resultados_cap}/bootstrapping/Pre_estimates_age_2017_rep`samp'.dta", replace

}


** Uniendo las estimaciones del bootstraping para poder sacar las medidas agregadas:


use "${resultados_cap}/bootstrapping/Pre_estimates_age_2017_rep1.dta", clear

foreach samp of numlist 2(1)1000 {

append using "${resultados_cap}/bootstrapping/Pre_estimates_age_2017_rep`samp'.dta"

}

** Haciendo las estimaciones de las prevalencias específicas y sensibles por estratos (sexo y edad)

table (age_group) (), statistic(mean p_str_sen )  statistic(p25 p_str_sen) statistic(p75 p_str_sen)
statistic(mean p_str_esp )  statistic(p25 p_str_esp) statistic(p75 p_str_esp) nototals
collect export "${resultados_cap}/Prevalencias_estrato_edad_2017.docx", as(docx) replace

********************************************************************************
******
**# Prevalencia por REGION
********************************************************************************
*******

foreach samp of numlist 1(1)1000 {

* Cargando la base
use "${resultados_cap}/bootstrapping/boots_Contributivo_n_3yrs_2017_rep`samp'.dta", clear

********************************************************************************
*******
** Estimando la prevalencia por sexo y grupo etario
```

Supporting file

```
merge 1:1 mujer region age_group using
"/home/javier/Documentos/Ca_pulmon/Bases_cap/Denominadores_BDUA.dta"
drop _merge

collapse (sum) def_sen def_esp denominador_2017, by(region)

gen cum_deno= sum(denominador_2017)
egen tot_denominador_2017 = max(cum_deno)
drop cum_deno

gen cumsen= sum(def_sen)
gen cumesp= sum(def_esp)

egen tot_sen = max(cumsen)
egen tot_esp = max(cumesp)

drop cumsen cumesp

gen p_str_sen= (def_sen/denominador_2017)*100000
gen p_str_esp= (def_esp/denominador_2017)*100000

gen p_total_sen = (tot_sen/tot_denominador_2017)*100000
gen p_total_esp = (tot_esp/tot_denominador_2017)*100000


save "${resultados_cap}/bootstrapping/Pre_estimates_region_2017_rep`samp'.dta", replace

}


** Uniendo las estimaciones del bootstraping para poder sacar las medidas agregadas:


use "${resultados_cap}/bootstrapping/Pre_estimates_region_2017_rep1.dta", clear

foreach samp of numlist 2(1)1000 {

append using "${resultados_cap}/bootstrapping/Pre_estimates_region_2017_rep`samp'.dta"

}

** Haciendo las estimaciones de las prevalencias específicas y sensibles por estratos (REGION)

table ( region) (), statistic(mean p_str_sen )  statistic(p25 p_str_sen) statistic(p75 p_str_sen)
statistic(mean p_str_esp )  statistic(p25 p_str_esp) statistic(p75 p_str_esp) nototals
collect export "${resultados_cap}/Prevalencias_estrato_region_2017.docx", as(docx) replace

** Haciendo la tabla de prevalencia general
```

table () (), statistic(mean p_str_sen) statistic(p25 p_str_sen) statistic(p75 p_str_sen) statistic(mean p_str_esp) statistic(p25 p_str_esp) statistic(p75 p_str_esp) nototals
collect export "${resultados_cap}/Prevalencias_total_2017.docx", as(docx) replace

****
log close

****
5_Prevalence_estimation_cap_2018_subsidized
****

* Opening the log file

clear all

*Log_files

global logs_cap /home/javier/Documentos/Ca_pulmon/1_log_files

*Ruta bases ORIGINALES:

global bases_ori /home/javier/Documentos/BASES_ORI

*Ruta bases CS:

global bases_cap /home/javier/Documentos/Ca_pulmon/Bases_cap

*Ruta resultados

global resultados_cap /home/javier/Documentos/Ca_pulmon/3_Resultados/Contributivo

*Abriendo el log

log using "${logs_cap}/Prevalence_estimation_2018-contributivo_bootstrapping.smcl", replace

****

**# Preparación de las bases para el bootstrapping

** Estableciendo los parametros para correr la identificación de sujetos con CAP

Supporting file

```
** Años para buscar los ID en la UPC

local anos_ID "2016 2017 2018"

local start_ID_year "2016"   // primer año necesita una estructura de appends diferente

local continua_ID_years "2017 2018" // años en los que se continua haciendo el append.

local final_ID_year "2018"   // año final en el cual se va a hacer la búsqueda de los casos para marcar los archivos.


** Años para buscar los servicios

local anos_serv "2016 2017 2018"

local start_serv_year "2016"   // primer año necesita una estructura de appends diferente

local continua_serv_years "2017 2018" // años en los que se continua haciendo el append.

local final_serv_year "2018"   // año final en el cual se va a hacer la búsqueda de servicios para marcar los archivos.


foreach samp of numlist 1(1)1000 {


** Cargando la BD de todos los pacientes en el periodo que tienen al menos un código de ca de pulmón

use "${bases_cap}/ca_ser_`start_serv_year'-`final_serv_year'_inicial.dta", replace


** Ajustando fecha de servicio

gen fx_servicio=date(FechaServicio, "YMD")

format fx_servicio %td

gen mes_servicio=mofd(fx_servicio)

format mes_servicio %tm

gen year_servicio=year(fx_servicio)
```

```
drop FechaServicio


** Excluyendo pacientes que no consumieron servicios en el último año de seguimiento (aplicando CRITERIO DE INCLUSIÓN EXPUESTOS# 1)----------------------------------------- INCLUSION#1

        bys PersonaBasicaID: egen max_servicio=max(year_servicio)

        keep if max_servicio==`final_serv_year'  // este código identifica aquellos pacientes que consiumieron servicios en el ultimo año de seguimiento


** Identificando todos los servicios con CIE10 de Ca de Pulmón 2)--------------------------------------------------------------------------------------- INCLUSION#2

        gen cie10_3d=DiagnosticoCD

        recast str3 cie10_3d, force

        gen ca_dx=1 if cie10_3d=="C34" /*| cie10_3d=="C33" | DiagnosticoCD=="D381"*/

        replace ca_dx=0 if ca_dx!=1


** Identificando CUPS de uso de servicios oncológicos 2)--------------------------------------------------------------------------------------------- INCLUSION#2

        gen     cups_ca=1 if ProcedimientoCD=="320002"

        replace cups_ca=1 if ProcedimientoCD=="320003"

        replace cups_ca=1 if ProcedimientoCD=="320201"

        replace cups_ca=1 if ProcedimientoCD=="322800"

        replace cups_ca=1 if ProcedimientoCD=="323100"

        replace cups_ca=1 if ProcedimientoCD=="324101"

        replace cups_ca=1 if ProcedimientoCD=="324102"

        replace cups_ca=1 if ProcedimientoCD=="324200"

        replace cups_ca=1 if ProcedimientoCD=="324201"

        replace cups_ca=1 if ProcedimientoCD=="324202"

        replace cups_ca=1 if ProcedimientoCD=="324203"

        replace cups_ca=1 if ProcedimientoCD=="324204"

        replace cups_ca=1 if ProcedimientoCD=="325100"
```

Supporting file

```
replace cups_ca=1 if ProcedimientoCD=="325101"
replace cups_ca=1 if ProcedimientoCD=="325102"
replace cups_ca=1 if ProcedimientoCD=="325200"
replace cups_ca=1 if ProcedimientoCD=="325201"
replace cups_ca=1 if ProcedimientoCD=="325202"
replace cups_ca=1 if ProcedimientoCD=="325300"
replace cups_ca=1 if ProcedimientoCD=="325301"
replace cups_ca=1 if ProcedimientoCD=="325302"
replace cups_ca=1 if ProcedimientoCD=="326101"
replace cups_ca=1 if ProcedimientoCD=="332203"
replace cups_ca=1 if ProcedimientoCD=="332204"
replace cups_ca=1 if ProcedimientoCD=="332206"
replace cups_ca=1 if ProcedimientoCD=="332207"
replace cups_ca=1 if ProcedimientoCD=="332401"
replace cups_ca=1 if ProcedimientoCD=="332500"
replace cups_ca=1 if ProcedimientoCD=="332501"
replace cups_ca=1 if ProcedimientoCD=="332601"
replace cups_ca=1 if ProcedimientoCD=="332701"
replace cups_ca=1 if ProcedimientoCD=="332702"
replace cups_ca=1 if ProcedimientoCD=="332703"
replace cups_ca=1 if ProcedimientoCD=="332704"
replace cups_ca=1 if ProcedimientoCD=="332801"
replace cups_ca=1 if ProcedimientoCD=="340200"
replace cups_ca=1 if ProcedimientoCD=="340201"
replace cups_ca=1 if ProcedimientoCD=="342100"
replace cups_ca=1 if ProcedimientoCD=="320001"
replace cups_ca=1 if ProcedimientoCD=="320202"
replace cups_ca=1 if ProcedimientoCD=="334400"
replace cups_ca=1 if ProcedimientoCD=="549004"
```

```
replace cups_ca=1 if ProcedimientoCD=="549011"
replace cups_ca=1 if ProcedimientoCD=="992501"
replace cups_ca=1 if ProcedimientoCD=="992502"
replace cups_ca=1 if ProcedimientoCD=="992503"
replace cups_ca=1 if ProcedimientoCD=="992504"
replace cups_ca=1 if ProcedimientoCD=="992505"
replace cups_ca=1 if ProcedimientoCD=="992510"
replace cups_ca=1 if ProcedimientoCD=="992506"
replace cups_ca=1 if ProcedimientoCD=="992507"
replace cups_ca=1 if ProcedimientoCD=="890287"
replace cups_ca=1 if ProcedimientoCD=="890387"
replace cups_ca=1 if ProcedimientoCD=="890487"
replace cups_ca=1 if ProcedimientoCD=="922506"
replace cups_ca=1 if ProcedimientoCD=="922800"
replace cups_ca=1 if ProcedimientoCD=="922801"
replace cups_ca=1 if ProcedimientoCD=="S22222"
replace cups_ca=0 if cups_ca!=1

** Identificando características de base

bys PersonaBasicaID: egen residencia=mode(MunicipioCD) if  year_servicio==`final_serv_year' | year_servicio==(`final_serv_year'-1), maxmode // ambitosprocedimientocd=="A" &

bys PersonaBasicaID: egen eps=mode(CodigoAdministradora) if year_servicio==`final_serv_year' | year_servicio==(`final_serv_year'-1), maxmode

gen f_nac=date(AnnoNacimiento, "YMD")
replace f_nac=date(AnnoNacimiento,"Y") if f_nac==.
format f_nac %td
drop AnnoNacimiento
```

Supporting file

```
bys PersonaBasicaID: egen sexo=mode(Sexo), maxmode


collapse (max) f_nac residencia (first) eps sexo (max) ca_dx cups_ca, by(PersonaBasicaID
mes_servicio)


collapse (max) f_nac residencia (first) eps sexo  (sum) ca_dx cups_ca, by(PersonaBasicaID)





***////////////////////////////////////////////////////////////////////////////////////////////////////////////////////
////
**********************************************************************************************
*************************
*** Linea para hacer el bootstraping


bsample, cluster(PersonaBasicaID) idcluster(PersonaBasicaID_s)


**********************************************************************************************
**************************
***////////////////////////////////////////////////////////////////////////////////////////////////////////////////////
////





** Sexo
gen mujer=1 if sexo=="F"
replace mujer=0 if sexo=="M"


drop if mujer==.
```

Supporting file

** Depto/Region

```
gen depto=int(residencia/1000)

label define depto 5 "Antioquia" 8 "Atlántico" 11 "Bogotá" 13 "Bolívar" 15 "Boyacá" 17 "Caldas" 18 "Caquetá" /*

*/ 19 "Cauca" 20 "Cesar" 23 "Córdoba" 25 "Cundinamarca" 27 "Choco" 41 "Huila" 44 "La Guajira" 47 "Magdalena" /*

*/ 50 "Meta" 52 "Nariño" 54 "N de Santander" 63 "Quindío" 66 "Risaralda" 68 "Santander" 70 "Sucre" 73 "Tolima" /*

*/ 76 "Valle del Cauca" 81 "Arauca" 85 "Casanare" 86 "Putumayo" 88 "San Andrés" 91 "Amazonas" 94 "Guainía" 95 "Guaviare" 97 "Vaupés" 99 "Vichada"

label values depto depto

gen region=1 if depto==8 | depto==13 | depto==20 | depto==23 | depto==44 | depto==47 | depto==70 | depto==88

replace region=2 if depto==11

replace region=3 if depto==5 | depto==17 | depto==18 | depto==41 | depto==63 | depto==66 | depto==73

replace region=4 if depto==15 | depto==25 | depto==50 | depto==54 | depto==68

replace region=5 if depto==91 | depto==81 | depto==85 | depto==86 | depto==88 | depto==94 | depto==95 | depto==97 | depto==99

replace region=6 if depto==76 | depto==19 | depto==27 | depto==52

label define region 1 "Atlántica" 2 "Bogotá DC" 3 "Central" 4 "Oriental" 5 "Orinoquía - Amazonía" 6 "Pacífica"

label values region region
```

*** Definiendo grupos de edad ***

```
gen age=(21185-f_nac)/365.25   // fecha 21185 1 de enero de 2018
keep if age>=20 & age<=110
gen age_group = 1 if age<45
replace age_group = 2 if age>=45 & age<65
replace age_group = 3 if age>=65

label define age_group 1 "20 a 44" 2 "45 a 64" 3 "65 o más"
label values age_group age_group

*************************************************************************


** Generar las definiciones


gen def_sen=1 if ca_dx>=4
gen def_esp=1 if ca_dx>=4 & cups_ca!=0



** Generando la base de datos


collapse (sum) def_sen def_esp, by(mujer region age_group)



save
"${resultados_cap}/bootstrapping/boots_Contributivo_n_3yrs_`final_serv_year'_rep`samp'.dta",
replace


}
```

Supporting file

```
******************************************************************************
******
**# Prevalencia por SEXO
******************************************************************************
*******

foreach samp of numlist 1(1)1000 {

* Cargando la base
use "${resultados_cap}/bootstrapping/boots_Contributivo_n_3yrs_2018_rep`samp'.dta", clear

******************************************************************************
*******
** Estimando la prevalencia por sexo y grupo etario

merge 1:1 mujer region age_group using
"/home/javier/Documentos/Ca_pulmon/Bases_cap/Denominadores_BDUA.dta"
drop _merge

collapse (sum) def_sen def_esp denominador_2018, by(mujer)

gen cum_deno= sum(denominador_2018)
egen tot_denominador_2018 = max(cum_deno)
drop cum_deno

gen cumsen= sum(def_sen)
gen cumesp= sum(def_esp)
```

```
egen tot_sen = max(cumsen)
egen tot_esp = max(cumesp)

drop cumsen cumesp

gen p_str_sen= (def_sen/denominador_2018)*100000
gen p_str_esp= (def_esp/denominador_2018)*100000

gen p_total_sen = (tot_sen/tot_denominador_2018)*100000
gen p_total_esp = (tot_esp/tot_denominador_2018)*100000


save "${resultados_cap}/bootstrapping/Pre_estimates_sex_2018_rep`samp'.dta", replace

}


** Uniendo las estimaciones del bootstraping para poder sacar las medidas agregadas:


use "${resultados_cap}/bootstrapping/Pre_estimates_sex_2018_rep1.dta", clear

foreach samp of numlist 2(1)1000 {

append using "${resultados_cap}/bootstrapping/Pre_estimates_sex_2018_rep`samp'.dta"

}

** Haciendo las estimaciones de las prevalencias específicas y sensibles por estratos (sexo y edad)
```

Supporting file

table (mujer) (), statistic(mean p_str_sen) statistic(p25 p_str_sen) statistic(p75 p_str_sen) statistic(mean p_str_esp) statistic(p25 p_str_esp) statistic(p75 p_str_esp) nototals

collect label levels mujer 0 "Hombre" 1 "Mujer", modify

collect export "${resultados_cap}/Prevalencias_estrato_sexo_2018.docx", as(docx) replace

****

**# Prevalencia por GRUPO ETARIO

****

foreach samp of numlist 1(1)1000 {

* Cargando la base

use "${resultados_cap}/bootstrapping/boots_Contributivo_n_3yrs_2018_rep`samp'.dta", clear

****

** Estimando la prevalencia por sexo y grupo etario

merge 1:1 mujer region age_group using "/home/javier/Documentos/Ca_pulmon/Bases_cap/Denominadores_BDUA.dta"

drop _merge

collapse (sum) def_sen def_esp denominador_2018, by(age_group)

gen cum_deno= sum(denominador_2018)

egen tot_denominador_2018 = max(cum_deno)

drop cum_deno

```
gen cumsen= sum(def_sen)

gen cumesp= sum(def_esp)


egen tot_sen = max(cumsen)

egen tot_esp = max(cumesp)


drop cumsen cumesp


gen p_str_sen= (def_sen/denominador_2018)*100000

gen p_str_esp= (def_esp/denominador_2018)*100000


gen p_total_sen = (tot_sen/tot_denominador_2018)*100000

gen p_total_esp = (tot_esp/tot_denominador_2018)*100000



save "${resultados_cap}/bootstrapping/Pre_estimates_age_2018_rep`samp'.dta", replace


}



** Uniendo las estimaciones del bootstraping para poder sacar las medidas agregadas:



use "${resultados_cap}/bootstrapping/Pre_estimates_age_2018_rep1.dta", clear


foreach samp of numlist 2(1)1000 {


append using "${resultados_cap}/bootstrapping/Pre_estimates_age_2018_rep`samp'.dta"
```

}

** Haciendo las estimaciones de las prevalencias específicas y sensibles por estratos (sexo y edad)

table (age_group) (), statistic(mean p_str_sen) statistic(p25 p_str_sen) statistic(p75 p_str_sen) statistic(mean p_str_esp) statistic(p25 p_str_esp) statistic(p75 p_str_esp) nototals

collect export "${resultados_cap}/Prevalencias_estrato_edad_2018.docx", as(docx) replace

****

**# Prevalencia por REGION

****

foreach samp of numlist 1(1)1000 {

* Cargando la base

use "${resultados_cap}/bootstrapping/boots_Contributivo_n_3yrs_`final_serv_year'_rep`samp'.dta", clear

****

** Estimando la prevalencia por sexo y grupo etario

merge 1:1 mujer region age_group using "/home/javier/Documentos/Ca_pulmon/Bases_cap/Denominadores_BDUA.dta"

drop _merge

collapse (sum) def_sen def_esp denominador_`final_serv_year', by(region)

```
gen cum_deno= sum(denominador_`final_serv_year')

egen tot_denominador_`final_serv_year' = max(cum_deno)

drop cum_deno


gen cumsen= sum(def_sen)

gen cumesp= sum(def_esp)


egen tot_sen = max(cumsen)

egen tot_esp = max(cumesp)


drop cumsen cumesp


gen p_str_sen= (def_sen/denominador_`final_serv_year')*100000

gen p_str_esp= (def_esp/denominador_`final_serv_year')*100000


gen p_total_sen = (tot_sen/tot_denominador_`final_serv_year')*100000

gen p_total_esp = (tot_esp/tot_denominador_`final_serv_year')*100000



save "${resultados_cap}/bootstrapping/Pre_estimates_region_`final_serv_year'_rep`samp'.dta",
replace


}



** Uniendo las estimaciones del bootstraping para poder sacar las medidas agregadas:
```

```
use "${resultados_cap}/bootstrapping/Pre_estimates_region_`final_serv_year'_rep1.dta", clear

foreach samp of numlist 2(1)1000 {

append using
"${resultados_cap}/bootstrapping/Pre_estimates_region_`final_serv_year'_rep`samp'.dta"

}

** Haciendo las estimaciones de las prevalencias específicas y sensibles por estratos (REGION)

table ( region) (), statistic(mean p_str_sen )  statistic(p25 p_str_sen) statistic(p75 p_str_sen)
statistic(mean p_str_esp )  statistic(p25 p_str_esp) statistic(p75 p_str_esp) nototals

collect export "${resultados_cap}/Prevalencias_estrato_region_`final_serv_year'.docx", as(docx)
replace

** Haciendo la tabla de prevalencia general

table () (), statistic(mean p_str_sen )  statistic(p25 p_str_sen) statistic(p75 p_str_sen)
statistic(mean p_str_esp )  statistic(p25 p_str_esp) statistic(p75 p_str_esp) nototals

collect export "${resultados_cap}/Prevalencias_total_`final_serv_year'.docx", as(docx) replace

*******************************************

log close
```

****
6_Prevalence_estimation_cap_2019_subsidized
****

```
* Opening the log file
	clear all
	*Log_files
	global logs_cap /home/javier/Documentos/Ca_pulmon/1_log_files
	*Ruta bases ORIGINALES:
	global bases_ori /home/javier/Documentos/BASES_ORI
	*Ruta bases CS:
	global bases_cap /home/javier/Documentos/Ca_pulmon/Bases_cap
```

Supporting file

```
	*Ruta resultados
	global resultados_cap /home/javier/Documentos/Ca_pulmon/3_Resultados/Contributivo
	*Abriendo el log
	log using "${logs_cap}/Prevalence_estimation_2019-contributivo_bootstrapping.smcl",
replace

**************************************************************************************************************
**# Preparación de las bases para el bootstrapping



** Estableciendo los parametros para correr la identificación de sujetos con CAP

	** Años para buscar los ID en la UPC
	local anos_ID "2017 2018 2019"
	local start_ID_year "2017"   // primer año necesita una estructura de appends diferente
	local continua_ID_years "2018 2019" // años en los que se continua haciendo el append.
	local final_ID_year "2019"   // año final en el cual se va a hacer la búsqueda de los casos
para marcar los archivos.


	** Años para buscar los servicios
	local anos_serv "2017 2018 2019"
	local start_serv_year "2017"   // primer año necesita una estructura de appends diferente
	local continua_serv_years "2018 2019" // años en los que se continua haciendo el append.
	local final_serv_year "2019"   // año final en el cual se va a hacer la búsqueda de servicios
para marcar los archivos.



foreach samp of numlist 1(1)1000 {


	** Cargando la BD de todos los pacientes en el periodo que tienen al menos un código de
ca de pulmón
	use "${bases_cap}/ca_ser_`start_serv_year'-`final_serv_year'_inicial.dta", replace

	** Ajustando fecha de servicio
	gen fx_servicio=date(FechaServicio, "YMD")
	format fx_servicio %td
	gen mes_servicio=mofd(fx_servicio)
	format mes_servicio %tm
	gen year_servicio=year(fx_servicio)
	drop FechaServicio

	** Excluyendo pacientes que no consumieron servicios en el último año de seguimiento
(aplicando CRITERIO DE INCLUSIÓN EXPUESTOS# 1)----------------------------------------- INCLUSION#1
```

```
bys PersonaBasicaID: egen max_servicio=max(year_servicio)
keep if max_servicio==`final_serv_year'  // este código identifica aquellos pacientes que consiumieron servicios en el ultimo año de seguimiento

** Identificando todos los servicios con CIE10 de Ca de Pulmón 2)-------------------------------------------------------------------------------------- INCLUSION#2
gen cie10_3d=DiagnosticoCD
recast str3 cie10_3d, force
gen ca_dx=1 if cie10_3d=="C34" /*| cie10_3d=="C33" | DiagnosticoCD=="D381"*/
replace ca_dx=0 if ca_dx!=1

** Identificando CUPS de uso de servicios oncológicos 2)-------------------------------------------------------------------------------------------- INCLUSION#2
gen     cups_ca=1 if ProcedimientoCD=="320002"
replace cups_ca=1 if ProcedimientoCD=="320003"
replace cups_ca=1 if ProcedimientoCD=="320201"
replace cups_ca=1 if ProcedimientoCD=="322800"
replace cups_ca=1 if ProcedimientoCD=="323100"
replace cups_ca=1 if ProcedimientoCD=="324101"
replace cups_ca=1 if ProcedimientoCD=="324102"
replace cups_ca=1 if ProcedimientoCD=="324200"
replace cups_ca=1 if ProcedimientoCD=="324201"
replace cups_ca=1 if ProcedimientoCD=="324202"
replace cups_ca=1 if ProcedimientoCD=="324203"
replace cups_ca=1 if ProcedimientoCD=="324204"
replace cups_ca=1 if ProcedimientoCD=="325100"
replace cups_ca=1 if ProcedimientoCD=="325101"
replace cups_ca=1 if ProcedimientoCD=="325102"
replace cups_ca=1 if ProcedimientoCD=="325200"
replace cups_ca=1 if ProcedimientoCD=="325201"
replace cups_ca=1 if ProcedimientoCD=="325202"
replace cups_ca=1 if ProcedimientoCD=="325300"
replace cups_ca=1 if ProcedimientoCD=="325301"
replace cups_ca=1 if ProcedimientoCD=="325302"
replace cups_ca=1 if ProcedimientoCD=="326101"
replace cups_ca=1 if ProcedimientoCD=="332203"
replace cups_ca=1 if ProcedimientoCD=="332204"
replace cups_ca=1 if ProcedimientoCD=="332206"
replace cups_ca=1 if ProcedimientoCD=="332207"
replace cups_ca=1 if ProcedimientoCD=="332401"
replace cups_ca=1 if ProcedimientoCD=="332500"
replace cups_ca=1 if ProcedimientoCD=="332501"
replace cups_ca=1 if ProcedimientoCD=="332601"
replace cups_ca=1 if ProcedimientoCD=="332701"
replace cups_ca=1 if ProcedimientoCD=="332702"
replace cups_ca=1 if ProcedimientoCD=="332703"
replace cups_ca=1 if ProcedimientoCD=="332704"
replace cups_ca=1 if ProcedimientoCD=="332801"
```

```
replace cups_ca=1 if ProcedimientoCD=="340200"
replace cups_ca=1 if ProcedimientoCD=="340201"
replace cups_ca=1 if ProcedimientoCD=="342100"
replace cups_ca=1 if ProcedimientoCD=="320001"
replace cups_ca=1 if ProcedimientoCD=="320202"
replace cups_ca=1 if ProcedimientoCD=="334400"
replace cups_ca=1 if ProcedimientoCD=="549004"
replace cups_ca=1 if ProcedimientoCD=="549011"
replace cups_ca=1 if ProcedimientoCD=="992501"
replace cups_ca=1 if ProcedimientoCD=="992502"
replace cups_ca=1 if ProcedimientoCD=="992503"
replace cups_ca=1 if ProcedimientoCD=="992504"
replace cups_ca=1 if ProcedimientoCD=="992505"
replace cups_ca=1 if ProcedimientoCD=="992510"
replace cups_ca=1 if ProcedimientoCD=="992506"
replace cups_ca=1 if ProcedimientoCD=="992507"
replace cups_ca=1 if ProcedimientoCD=="890287"
replace cups_ca=1 if ProcedimientoCD=="890387"
replace cups_ca=1 if ProcedimientoCD=="890487"
replace cups_ca=1 if ProcedimientoCD=="922506"
replace cups_ca=1 if ProcedimientoCD=="922800"
replace cups_ca=1 if ProcedimientoCD=="922801"
replace cups_ca=1 if ProcedimientoCD=="S22222"
replace cups_ca=0 if cups_ca!=1

** Identificando características de base

bys PersonaBasicaID: egen residencia=mode(MunicipioCD) if  year_servicio==`final_serv_year' | year_servicio==(`final_serv_year'-1), maxmode // ambitosprocedimientocd=="A" &
bys PersonaBasicaID: egen eps=mode(CodigoAdministradora) if year_servicio==`final_serv_year' | year_servicio==(`final_serv_year'-1), maxmode

gen f_nac=date(AnnoNacimiento, "YMD")
replace f_nac=date(AnnoNacimiento,"Y") if f_nac==.
format f_nac %td
drop AnnoNacimiento

bys PersonaBasicaID: egen sexo=mode(Sexo), maxmode

collapse (max) f_nac residencia (first) eps sexo (max) ca_dx cups_ca, by(PersonaBasicaID mes_servicio)

collapse (max) f_nac residencia (first) eps sexo  (sum) ca_dx cups_ca, by(PersonaBasicaID)
```

Supporting file

```
***/////////////////////////////////////////////////////////////////////////////////////////////
////
*********************************************************************************
*************************
*** Linea para hacer el bootstraping

bsample, cluster(PersonaBasicaID) idcluster(PersonaBasicaID_s)

*********************************************************************************
**************************
***/////////////////////////////////////////////////////////////////////////////////////////////
////




** Sexo
gen mujer=1 if sexo=="F"
replace mujer=0 if sexo=="M"

drop if mujer==.

** Depto/Region

gen depto=int(residencia/1000)

label define depto 5 "Antioquia" 8 "Atlántico" 11 "Bogotá" 13 "Bolívar" 15 "Boyacá" 17 "Caldas" 18
"Caquetá" /*
*/ 19 "Cauca" 20 "Cesar" 23 "Córdoba" 25 "Cundinamarca" 27 "Choco" 41 "Huila" 44 "La Guajira"
47 "Magdalena" /*
*/ 50 "Meta" 52 "Nariño" 54 "N de Santander" 63 "Quindío" 66 "Risaralda" 68 "Santander" 70
"Sucre" 73 "Tolima" /*
*/ 76 "Valle del Cauca" 81 "Arauca" 85 "Casanare" 86 "Putumayo" 88 "San Andrés" 91 "Amazonas"
94 "Guainía" 95 "Guaviare" 97 "Vaupés" 99 "Vichada"

label values depto depto

gen region=1 if depto==8 | depto==13 | depto==20 | depto==23 | depto==44 | depto==47 |
depto==70 | depto==88
replace region=2 if depto==11
replace region=3 if depto==5 | depto==17 | depto==18 | depto==41 | depto==63 | depto==66 |
depto==73
replace region=4 if depto==15 | depto==25 | depto==50 | depto==54 | depto==68
replace region=5 if depto==91 | depto==81 | depto==85 | depto==86 | depto==88 | depto==94 |
depto==95 | depto==97 | depto==99
replace region=6 if depto==76 | depto==19 | depto==27 | depto==52
```

```
label define region 1 "Atlántica" 2 "Bogotá DC" 3 "Central" 4 "Oriental" 5 "Orinoquía - Amazonía" 6
"Pacífica"
label values region region


*** Definiendo grupos de edad ***

gen age=(21550-f_nac)/365.25   // fecha 21550 1 de enero de 2019
keep if age>=20 & age<=110
gen age_group = 1 if age<45
replace age_group = 2 if age>=45 & age<65
replace age_group = 3 if age>=65

label define age_group 1 "20 a 44" 2 "45 a 64" 3 "65 o más"
label values age_group age_group

**********************************************************************


** Generar las definiciones

gen def_sen=1 if ca_dx>=4
gen def_esp=1 if ca_dx>=4 & cups_ca!=0


** Generando la base de datos

collapse (sum) def_sen def_esp, by(mujer region age_group)


save
"${resultados_cap}/bootstrapping/boots_Contributivo_n_3yrs_`final_serv_year'_rep`samp'.dta",
replace

}


*****************************************************************************
******
**# Prevalencia por SEXO
*****************************************************************************
*******

foreach samp of numlist 1(1)1000 {

* Cargando la base
use "${resultados_cap}/bootstrapping/boots_Contributivo_n_3yrs_2019_rep`samp'.dta", clear
```

Supporting file

```
*******************************************************************************
*******
** Estimando la prevalencia por sexo y grupo etario

merge 1:1 mujer region age_group using
"/home/javier/Documentos/Ca_pulmon/Bases_cap/Denominadores_BDUA.dta"
drop _merge

collapse (sum) def_sen def_esp denominador_2019, by(mujer)

gen cum_deno= sum(denominador_2019)
egen tot_denominador_2019 = max(cum_deno)
drop cum_deno

gen cumsen= sum(def_sen)
gen cumesp= sum(def_esp)

egen tot_sen = max(cumsen)
egen tot_esp = max(cumesp)

drop cumsen cumesp

gen p_str_sen= (def_sen/denominador_2019)*100000
gen p_str_esp= (def_esp/denominador_2019)*100000

gen p_total_sen = (tot_sen/tot_denominador_2019)*100000
gen p_total_esp = (tot_esp/tot_denominador_2019)*100000


save "${resultados_cap}/bootstrapping/Pre_estimates_sex_2019_rep`samp'.dta", replace

}


** Uniendo las estimaciones del bootstraping para poder sacar las medidas agregadas:


use "${resultados_cap}/bootstrapping/Pre_estimates_sex_2019_rep1.dta", clear

foreach samp of numlist 2(1)1000 {

append using "${resultados_cap}/bootstrapping/Pre_estimates_sex_2019_rep`samp'.dta"

}

** Haciendo las estimaciones de las prevalencias específicas y sensibles por estratos (sexo y edad)
```

```
table ( mujer) (), statistic(mean p_str_sen )  statistic(p25 p_str_sen) statistic(p75 p_str_sen)
statistic(mean p_str_esp )  statistic(p25 p_str_esp) statistic(p75 p_str_esp) nototals
collect label levels mujer 0 "Hombre" 1 "Mujer", modify
collect export "${resultados_cap}/Prevalencias_estrato_sexo_2019.docx", as(docx) replace

******************************************************************************
******
**# Prevalencia por GRUPO ETARIO
******************************************************************************
*******

foreach samp of numlist 1(1)1000 {

* Cargando la base
use "${resultados_cap}/bootstrapping/boots_Contributivo_n_3yrs_2019_rep`samp'.dta", clear

******************************************************************************
*******
** Estimando la prevalencia por sexo y grupo etario

merge 1:1 mujer region age_group using
"/home/javier/Documentos/Ca_pulmon/Bases_cap/Denominadores_BDUA.dta"
drop _merge

collapse (sum) def_sen def_esp denominador_2019, by(age_group)

gen cum_deno= sum(denominador_2019)
egen tot_denominador_2019 = max(cum_deno)
drop cum_deno

gen cumsen= sum(def_sen)
gen cumesp= sum(def_esp)

egen tot_sen = max(cumsen)
egen tot_esp = max(cumesp)

drop cumsen cumesp

gen p_str_sen= (def_sen/denominador_2019)*100000
gen p_str_esp= (def_esp/denominador_2019)*100000

gen p_total_sen = (tot_sen/tot_denominador_2019)*100000
gen p_total_esp = (tot_esp/tot_denominador_2019)*100000


save "${resultados_cap}/bootstrapping/Pre_estimates_age_2019_rep`samp'.dta", replace

}
```

Supporting file

```
** Uniendo las estimaciones del bootstraping para poder sacar las medidas agregadas:

use "${resultados_cap}/bootstrapping/Pre_estimates_age_2019_rep1.dta", clear

foreach samp of numlist 2(1)1000 {

append using "${resultados_cap}/bootstrapping/Pre_estimates_age_2019_rep`samp'.dta"

}

** Haciendo las estimaciones de las prevalencias específicas y sensibles por estratos (sexo y edad)

table (age_group) (), statistic(mean p_str_sen )  statistic(p25 p_str_sen) statistic(p75 p_str_sen)
statistic(mean p_str_esp )  statistic(p25 p_str_esp) statistic(p75 p_str_esp) nototals
collect export "${resultados_cap}/Prevalencias_estrato_edad_2019.docx", as(docx) replace

*******************************************************************************
******
**# Prevalencia por REGION
*******************************************************************************
*******

foreach samp of numlist 1(1)1000 {

* Cargando la base
use
"${resultados_cap}/bootstrapping/boots_Contributivo_n_3yrs_`final_serv_year'_rep`samp'.dta",
clear

*******************************************************************************
*******
** Estimando la prevalencia por sexo y grupo etario

merge 1:1 mujer region age_group using
"/home/javier/Documentos/Ca_pulmon/Bases_cap/Denominadores_BDUA.dta"
drop _merge

collapse (sum) def_sen def_esp denominador_`final_serv_year', by(region)

gen cum_deno= sum(denominador_`final_serv_year')
egen tot_denominador_`final_serv_year' = max(cum_deno)
drop cum_deno

gen cumsen= sum(def_sen)
gen cumesp= sum(def_esp)
```

```
egen tot_sen = max(cumsen)
egen tot_esp = max(cumesp)

drop cumsen cumesp

gen p_str_sen= (def_sen/denominador_`final_serv_year')*100000
gen p_str_esp= (def_esp/denominador_`final_serv_year')*100000

gen p_total_sen = (tot_sen/tot_denominador_`final_serv_year')*100000
gen p_total_esp = (tot_esp/tot_denominador_`final_serv_year')*100000


save "${resultados_cap}/bootstrapping/Pre_estimates_region_`final_serv_year'_rep`samp'.dta",
replace

}


** Uniendo las estimaciones del bootstraping para poder sacar las medidas agregadas:


use "${resultados_cap}/bootstrapping/Pre_estimates_region_`final_serv_year'_rep1.dta", clear

foreach samp of numlist 2(1)1000 {

append using
"${resultados_cap}/bootstrapping/Pre_estimates_region_`final_serv_year'_rep`samp'.dta"

}

** Haciendo las estimaciones de las prevalencias específicas y sensibles por estratos (REGION)

table ( region) (), statistic(mean p_str_sen )  statistic(p25 p_str_sen) statistic(p75 p_str_sen)
statistic(mean p_str_esp )  statistic(p25 p_str_esp) statistic(p75 p_str_esp) nototals
collect export "${resultados_cap}/Prevalencias_estrato_region_`final_serv_year'.docx", as(docx)
replace

** Haciendo la tabla de prevalencia general
table () (), statistic(mean p_str_sen )  statistic(p25 p_str_sen) statistic(p75 p_str_sen)
statistic(mean p_str_esp )  statistic(p25 p_str_esp) statistic(p75 p_str_esp) nototals
collect export "${resultados_cap}/Prevalencias_total_`final_serv_year'.docx", as(docx) replace


*****************
log close
```
