# Supplementary material for: Prevalence of lung cancer in Colombia and a new diagnostic algorithm using health administrative databases: A real-world evidence study
Source: PLoS One. 2023 Mar 10;18(3):e0269079. doi: 10.1371/journal.pone.0269079 (PMC10004567; doi:10.1371/journal.pone.0269079)
Supplement: Attachment — Submitted filename: PlosOne1.docx [file pone.0269079.s002.docx]

The authors used an electronic algorithm to identify lung cancer prevalent patients in Colombia using administrative claims databases, as well as to estimate prevalence rates by age, sex, and geographic region. The topic is relevant, and the manuscript is well written. However, there are some major and minor issues that must be addressed.

The introduction is lengthy, and some sentences are repeated in the discussion and conclusion (e.g., last sentences, lines 81,82).

Despite the fact that the authors claim that the study was approved by an ethics committee, it is unclear what level of personal information was accessed (name, age, etc.). Please specify whether the information accessed was personal or non-identifiable. This point leads me to suspect that there is probably duplicated information. How many times can the same patient's information be entered into the database? Is there a unique identification number? Please elaborate on the strategies for removing repeat patients from the system. If none, acknowledge that over the limitations

Lines 87-94 - Please clarify the representativeness and coverage of the UPC and BDUA databases in relation to the entire Colombian population. It is also necessary to provide an estimated time for the system to register these medical claims.

Although the author stated in line 79 that the main goal of this article was to "...*develop an electronic algorithm*...", there is no information on how these algorithms were developed. The statistical methods used may be of interest to the readers; if they have already been published, please include references. If not, include them in the supplementary material, along with the .codes (.R code, Stata, Python, and so on). Please consider also including a description of the parameter used to fit these algorithms, methods, and, most importantly, the parameters measured to estimate the sensitivity and specificity, as well as the “gold standards”, include them either in the Methods or supplementary material.

Please explain why only 2017 estimates were provided. Consider providing prevalence estimates by year. It is also critical to include confidence intervals for your estimates, for example, Bootstrap techniques (talk with a colleague statistician to help with that).

Globocan provides cancer prevalence estimates in 5-year, including all (diagnosed, in treatment, and cured), for example, the number of people alive today who were diagnosed with cancer in the previous five years. This fact poses some difficulties, and some comparisons made by the authors are invalid. Please, consider discussing if the dataset can identify when these patients were diagnosed with cancer for the first time. Furthermore, it is unclear whether these patients were alive or dead in 2017. Deaths should not be accounted for.

Because lung cancer is a common site of metastasis, the author may want to address this issue. Is it possible to tell which patients have primary site cancer and which have metastasis from another site? If not, how should this affect the projections?

Some minor issues:

Line 179 – This sentence is redundant; consider removing it.

Line 180 – Is there any evidence that these samples are chosen at random? If this is the case, the estimates should be unaffected.

Line 207 - Consider updating the estimates for 2020 (GLOBOCAN)

Line 218 - This sentence is unclear.
